# Differential Protective Roles of c-Jun N-terminal Kinase-2 in Nonparenchymal Liver Cells and Hepatocytes During Cholestasis

**DOI:** 10.1016/j.jcmgh.2025.101588

**Published:** 2025-07-16

**Authors:** Mohamed Ramadan Mohamed, Gang Zhao, Ines Volkert, Antonio Molinaro, Carolin V. Schneider, Pavel Strnad, Jan G. Hengstler, Chi Xu, Roger J. Davis, Kai Markus Schneider, Francisco Javier Cubero, Christian Trautwein

**Affiliations:** 1 Department of Internal Medicine III, University Hospital, RWTH Aachen, Germany; 2 Center of Gallstone Disease, Shanghai East Hospital, Tongji University School of Medicine, Shanghai, China; 3 Department of Clinical and Molecular Medicine, Sahlgrenska Academy, Wallenberg Centre for Molecular and Translational Medicine, University of Gothenburg, Gothenburg, Sweden; 4 Department of Toxicology, Leibniz Research Centre for Working Environment and Human Factors (IfADo), Dortmund, Germany; 5 Department of Immunology, Ophthalmology, and ORL, Complutense University School of Medicine, Madrid, Spain; 6 Howard Hughes Medical Institute and University of Massachusetts Medical School, Worcester, Massachusetts; 7 Centre for Biomedical Research, Network on Liver and Digestive Diseases (CIBEREHD), Madrid, Spain; 8 Health Research Institute Gregorio Marañón (IiSGM), Madrid, Spain; 9 Institute for Research in Neurochemistry (IUIN), Complutense University of Madrid, Madrid, Spain; 10 Liver Center Stuttgart, Clinic for Gastroenterology, Klinikum Stuttgart, Germany

**Keywords:** Bile Acids, c-Jun N-terminal Kinase, Cholestasis, Hepatocytes, Nonparenchymal Cells

## Abstract

**Background & Aims:**

Cell-type-specific mechanisms are crucial in determining liver disease progression (eg, during cholestasis). Here, we defined the role of c-Jun N-terminal kinases 2 (JNK2) in cholestasis. Specifically, we studied differential JNK2 functions for controlling bile acid (BA) homeostasis and inflammation.

**Methods:**

Mice lacking *Jnk2* function, either specifically in hepatocytes (*Jnk2*^*Δhepa*^), globally (*Jnk2*^*-/-*^), or in combination with hepatocyte-specific *Jnk1* deletion (*Jnk1*^*Δhepa*^*/2*^*-/-*^*),* were subjected to bile duct ligation (BDL). Parameters of liver injury, inflammation, BA composition, synthesis and transport, and fibrosis were studied. Additionally, bone marrow transplantation (BMT) experiments were performed. Furthermore, primary cultured hepatocytes were incubated with hyper-interleukin (IL)6, and hepatic BA transporters were analyzed.

**Results:**

*Jnk2*^*-/-*^ mice triggered increased liver injury, inflammation, and fibrosis compared with WT and *Jnk2*^*Δhepa*^ mice after BDL. However, *Jnk1*^*Δhepa*^*/2*^*-/-*^ mice exhibited an aggravated phenotype compared with *Jnk2*^*-/-*^ livers as indicated by enhanced hepatic damage, ductular reaction, inflammatory response, and fibrogenesis. BMT experiments excluded BM-derived cells and indicated that nonparenchymal liver cells (NPCs) play a critical role in driving the *Jnk1*^*Δhepa*^*/2*^*-/-*^-dependent severe phenotype after BDL. Furthermore, in vivo and in vitro analysis demonstrated a pivotal role for JNK signaling in hepatocytes in the regulation of BA homeostasis and transport.

**Conclusions:**

Besides in hepatocytes, JNK2 in NPCs—but not in BM-derived cells—confers protection during cholestasis. After BDL, a JNK2-dependent mechanism directs the inflammatory response involved in regulating biliary transporters. Hence, our data define JNK2 as a critical target during cholestasis in NPCs.

## Graphical abstract

**Figure:**
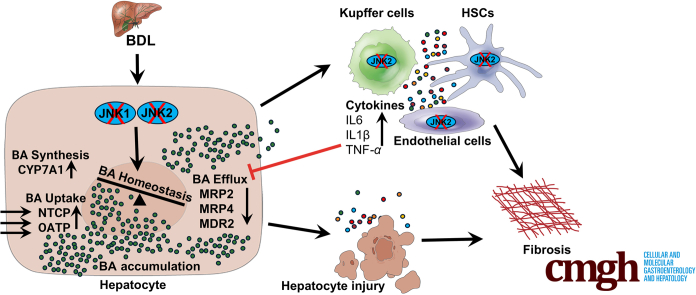

**Summary**

Our experiments reveal that c-Jun N-terminal kinase (JNK)2 exerts distinct, cell-type-specific roles during cholestasis. In nonparenchymal liver cells, but not in bone marrow-derived cells, JNK2 modulates the magnitude of the inflammatory response, whereas in hepatocytes, JNK1/2 prevents cholestatic liver injury by preserving bile acid homeostasis. Consistently, our data underscore the disruption of bile acid homeostasis in cholestatic patients and define differential JNK2-dependent functions during cholestasis.

Cholestatic liver injury is a pathologic condition triggered by impaired or blocked bile acid (BA) flow from the liver to the gut. Different mechanisms can lead to cholestasis. This includes mechanical obstruction of bile ducts by gallstones and tumors, immune-mediated biliary cholestasis, genetic defects of canalicular BA transporters, or drug-induced liver toxicity. As a result, bile salts and other toxic compounds accumulate in the liver, triggering an inflammatory response associated with increased cytokine/chemokine release, hepatic damage, ductular reaction, and subsequently progression to cirrhosis.^1^^,^^2^ Here, BA regulation and composition play a pivotal role as they mediate liver damage and, consecutively, disease progression.^3^^,^^4^

BAs are exclusively synthesized in hepatocytes from cholesterol mainly by cholesterin-7α-Hydroxylase (CYP7A1). However, to maintain a low level of toxic BAs within hepatocytes, an intact BA homeostasis coordinated by hepatobiliary transporters located within membranes of hepatocytes, cholangiocytes, and enterocytes is mandatory. These transporters are responsible for the efflux and the uptake of BAs and other toxic compounds from and into hepatocytes.^5^ The bile salt export pump (BSEP) releases most BA species from hepatocytes to bile canaliculi, whereas the ABC transporters, including multidrug resistance protein (MRP) family and multidrug resistance (MDR) family, are responsible for secreting sulfated BAs, unusual BAs, and other toxic compounds. The BA uptake into hepatocytes from the bloodstream is mainly regulated by Na^+^-taurocholate co-transporting polypeptide (NTCP) and the organic anion transporting polypeptides (OATP) being expressed at the basolateral membrane of hepatocytes.^5^ Disturbances in BA homeostasis and transporters have been reported in patients with cholestasis.^6^, ^7^, ^8^, ^9^, ^10^, ^11^

C-Jun N-terminal kinases (JNKs) belong to the mitogen-activated protein kinase (MAPK) family and have 3 different isoforms, namely *Jnk1* (*Mapk8*), *Jnk2* (*Mapk9*), and *Jnk3* (*Mapk10*). All mammalian cells, including hepatocytes, express *Jnk1* and *Jnk2*, whereas *Jnk3* is exclusively expressed in brain, heart, and testis tissue.^12^^,^^13^ Although JNK1 and JNK2 have largely similar and overlapping functions, these closely related proteins showed different and even contradictory effects during cholestasis.^14^ Hepatocytes play a pivotal role in BA synthesis, leading to the initial assumption that JNK1 or JNK2 functions are limited to these cells. Surprisingly, hepatocyte-specific deletion of either JNK1 or JNK2 does not affect cholestasis progression, suggesting a function of JNK genes in other cells (eg, hepatic stellate cells), as we previously reported for JNK1.^15^^,^^16^ In contrast, combined hepatocyte-specific deletion of both *Jnk1* and *Jnk2* aggravated disease progression after bile duct ligation (BDL)-induced cholestasis.^15^ Moreover, mice with constitutive ablation of *Jnk2* (*Jnk2*^*-/-*^) displayed increased cholestasis-induced fibrosis, 21 days after BDL, but the role of *Jnk2* for maintaining BA homeostasis was not addressed.^14^ Together, these results suggest that JNK2 has a specific role in cells other than hepatocytes during cholestasis.

In the present study, we applied the BDL model to better define the cell-specific role of JNK2 during cholestasis. We show that *Jnk2* in nonparenchymal cells (NPCs)—but not bone marrow (BM)-derived immune cells—is required to prevent liver injury during cholestasis. Specifically, we found that JNK2 regulates the inflammatory response in NPCs during cholestasis.

## Results

### Differential Function of *Jnk2* in Hepatocytes and NPCs

**Figure 1:**
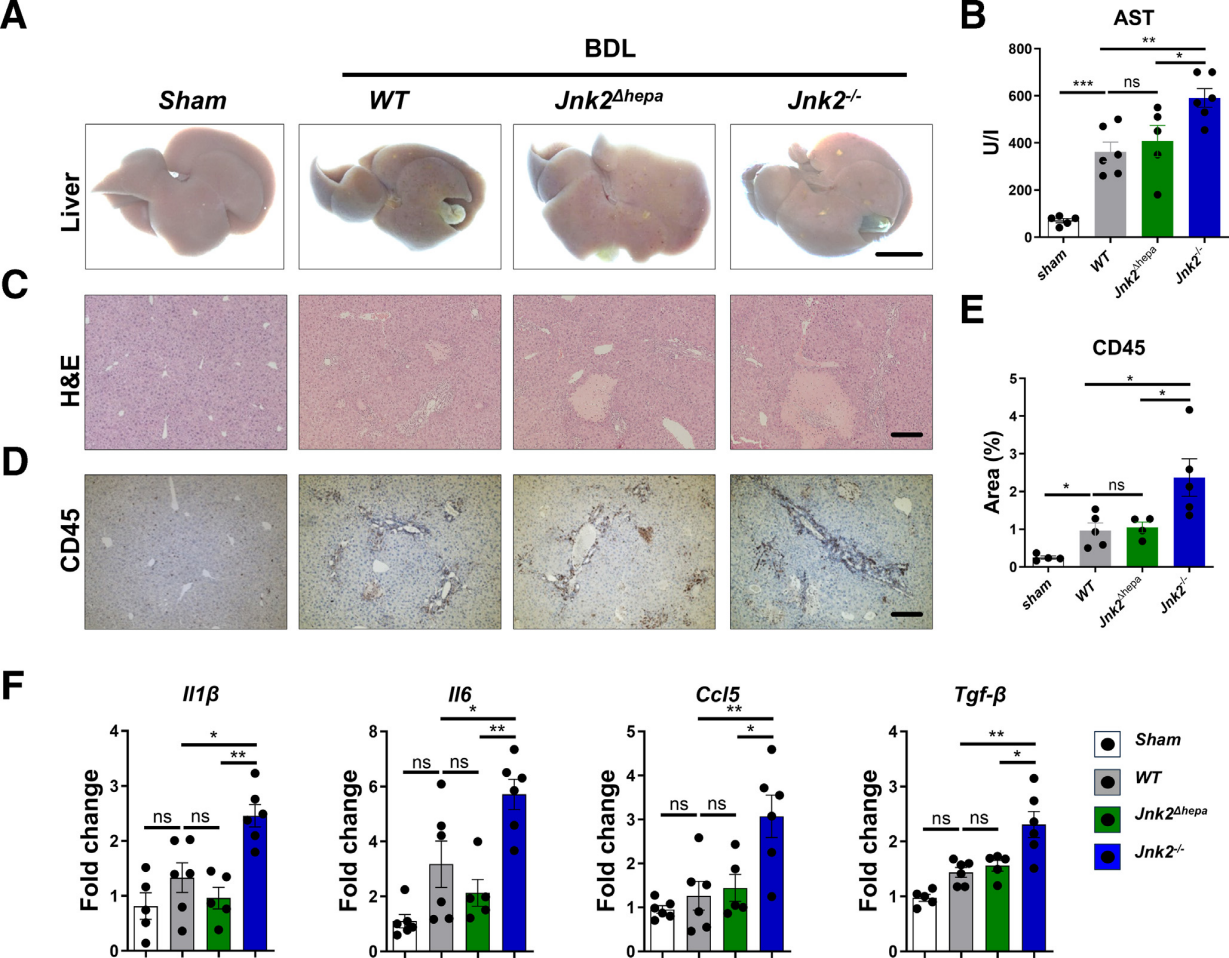
***Jnk2* deficiency in nonhepatocytes enhances liver injury and inflammation after BDL.** (*A–F*) *Jnk2*^*f/f*^ (WT), *Jnk2*^*Δhepa*^ and *Jnk2*^−/−^ mice were subjected to BDL for 28 days. Sham-operated mice are used as controls. (*A*) Representative macroscopic view of the livers (Scale bars, 1 cm). (*B*) Serum AST levels. (*C*) Representative H&E-stained liver sections (Scale bars, 100 μm). (*D–E*) Representative IHC and quantification of CD45 staining in liver sections of the same mice (Scale bar, 100 μm). (*F*) Hepatic expression of *Il1β, Il6, Ccl5,* and *Tgf-β* mRNA was determined by qRT-PCR and normalized to *Gapdh* mRNA level. Data are shown as means ± SEM from 5 to 6 mice per group (ns, non-significant; ∗*P* < .05, *∗∗P* < .01; 1-way ANOVA with Bonferroni’s multiple comparisons test).

**Figure 2:**
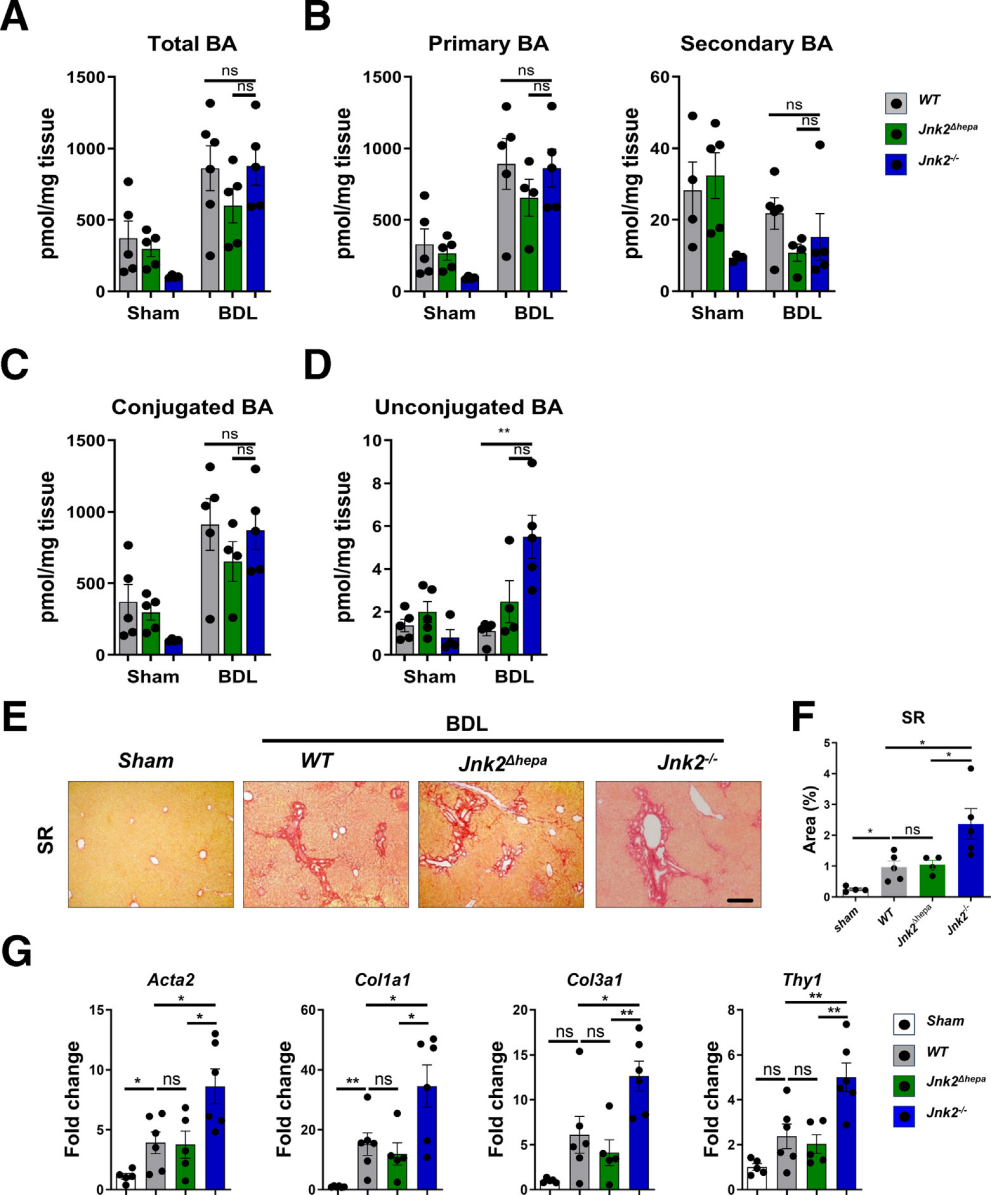
**Increased hepatic unconjugated BA content and fibrosis in response to *Jnk2* deletion in nonhepatocyte cells after BDL.** (*A–G*) *Jnk2*^*f/f*^ (WT), *Jnk2*^*Δhepa*^ and *Jnk2*^−/−^ mice were subjected to BDL for 28 days. Sham-operated mice are used as controls. Hepatic BA content in *Jnk2*^*f/f*^ (WT), *Jnk2*^*Δhepa*^ and *Jnk2*^−/−^ mice after BDL. (*A*) Total, (*B*) primary, secondary, (*C*) conjugated and (*D*) unconjugated BA concentration in (*Jnk2*^*f/f*^ (WT), *Jnk2*^*Δhepa*^ and *Jnk2*^−/−^; n = 5 per genotype) in the livers of mice after BDL or sham surgery. (*E–F*) Imaging and quantification of SR staining in sections of liver tissue (Scale bars, 100 μm). (*G*) Hepatic *Acta2, Col1a1, Col3a1,* and *Thy1* mRNA expression in liver derived from the same mice 28 days after BDL was determined by qRT-PCR and normalized to *Gapdh* mRNA level. Data are shown as means ± SEM from 5 to 6 mice per group (ns, non-significant; *∗P* < .05, *∗∗P* < .01, 1-way ANOVA with Bonferroni’s multiple comparisons test).

Our previous results demonstrated that a *Jnk1* or *Jnk2* hepatocyte-specific knockout has no impact on acute or chronic cholestatic liver injury, whereas *Jnk1* in hepatic stellate cells (HSCs) is crucial for their activation and, consequently, fibrosis progression.^15^^,^^16^ BDL in constitutive *Jnk2* knockout mice (*Jnk2*^*-/-*^) triggered significant liver injury and fibrosis compared with hepatocyte-specific *Jnk2* (*Jnk2*^*Δhepa*^) and wild-type floxed (WT) mice. As shown previously,^15^ BDL in WT and *Jnk2*^*Δhepa*^ mice induced macroscopically visible bile infarcts on the liver surface (Figure 1*A*). This was associated with increased serum aspartate aminotransferase (AST) (Figure 1*B*), necrotic areas, and inflammation surrounding the periportal area as shown by hematoxylin and eosin (H&E) and CD45 staining, respectively, but without significant differences between both strains (Figure 1*C–E*). In contrast, the phenotype was more pronounced in *Jnk2*^*-/-*^*-*BDL-treated mice (Figure 1*A–E*). Furthermore, BDL in *Jnk2*^*-/-*^*-*mice enhanced expression of pro-inflammatory cytokines/chemokines including *interleukin (Il)6*, *Il1β, CC-chemokine ligand 5 (Ccl5),* and *transforming growth factor beta (Tgf-β)* (Figure 1*F*). No significant changes in total hepatic BA composition, primary, secondary, or conjugated BAs were found between the studied mouse groups (Figure 2*A–C*), whereas unconjugated BA composition was significantly higher in *Jnk2*^*-/-*^ than WT livers after BDL (Figure 2*D*). Moreover, Sirius Red (SR) staining (indicative of hepatic fibrosis) increased in WT and *Jnk2*^*Δhepa*^ mice with BDL compared with sham-operated controls. However, in *Jnk2*^*-/-*^ livers, significantly enhanced fibrosis was evident (Figure 2*E–F*). This was strengthened by upregulation of fibrosis-related genes, including α-smooth muscle actin (*Acta2), collagen (Col) 1a1, Col3a1* and thymocyte differentiation antigen *(Thy1)* (Figure 2*G*). These data suggested that *Jnk2* in cells other than hepatocytes confer protection during chronic cholestatic liver injury.

### *Jnk2* in Nonhepatic Cells Protects From Liver Injury After BDL

**Figure 3:**
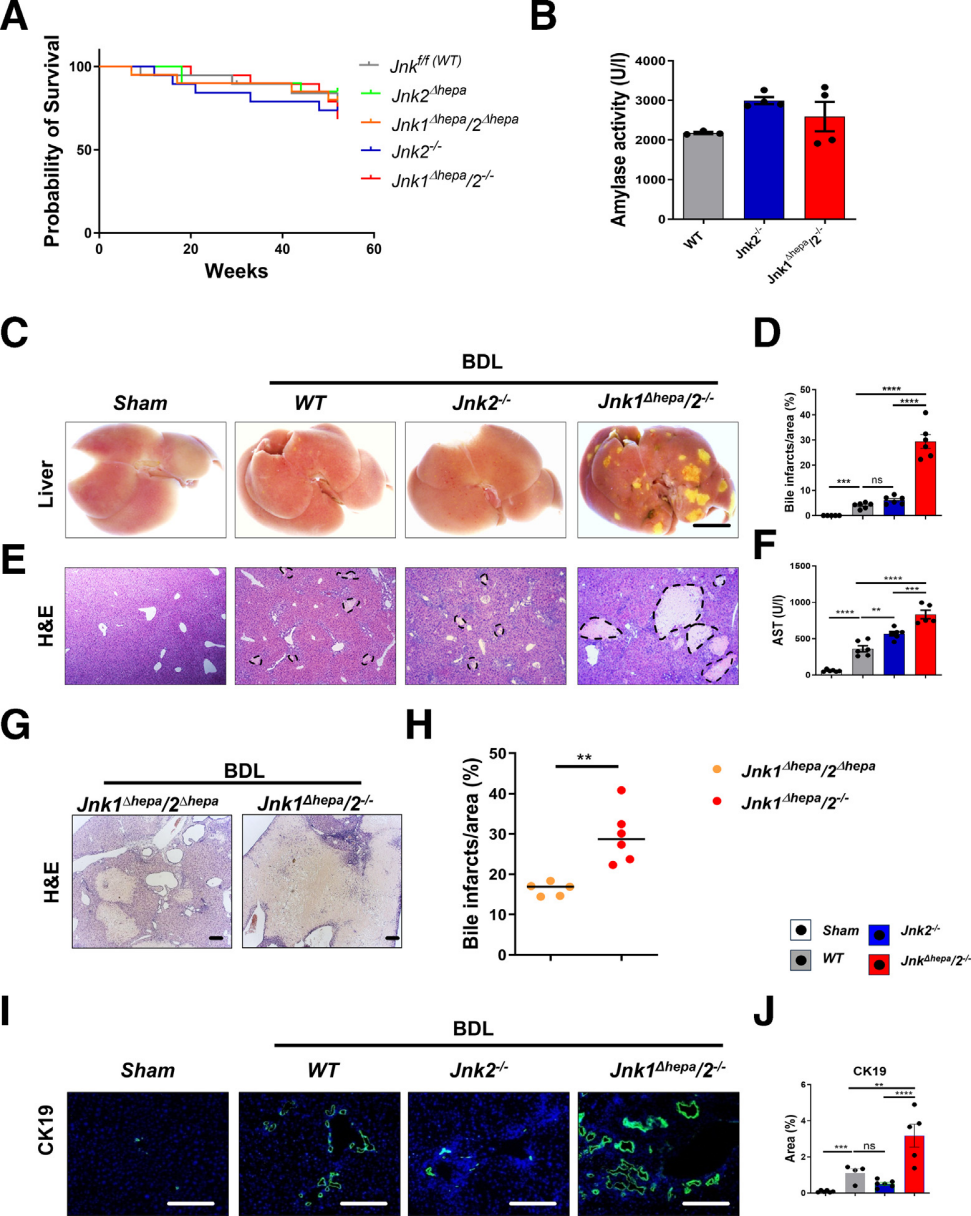
***Jnk2* in NPCs and combined hepatocytic *Jnk1* and *Jnk2* cooperate to protect against BDL-induced hepatic injury.** (*A*) Kaplan-Meier analysis of the survival of WT, *Jnk2*^*Δhepa*^, *Jnk2*^*-/-*^*, Jnk1*^*Δhepa*^*/2*^*Δhepa*^*, and Jnk1*^*Δhepa*^*/2*^*-/-*^ mice (n = 8–12) aged up to 52 weeks. (*B*) Serum amylase activity was measured in *Jnk*^*f/f*^ (WT), *Jnk2*^−/−^, and *Jnk1*^*Δhepa*^/*2*^−/−^ mice (n = 4–5 per group). (*C–J*) *Jnk*^*f/f*^ (WT), *Jnk2*^−/−^, *Jnk1*^*Δhepa*^/*2*^*Δhepa*^, and *Jnk1*^*Δhepa*^/*2*^−/−^ mice were subjected to BDL for 28 days. Sham-operated mice are used as controls (n = 5–6 per genotype). (*C*) Representative macroscopic view of the livers (Scale bars, 1 cm). (*D*) Representative H&E-stained liver sections from the same (Scale bar, 100 μm). (*E*) Quantification of bile infarct area. (*F*) Representative H&E-stained liver sections from *Jnk1*^*Δhepa*^/*2*^*Δhepa*^ and *Jnk1*^*Δhepa*^/*2*^−/−^ mice at day 28 after BDL (Scale bars, 100 μm). (*G*) Quantification of bile infarct area from *Jnk1*^*Δhepa*^/*2*^*Δhepa*^ and *Jnk1*^*Δhepa*^/*2*^−/−^ 28 days after BDL (n = 5–6 animals per genotype). (*H*) Serum AST levels. (*I*) Representative IF and quantification of CK19 staining in liver sections derived from WT*, Jnk2*^−/−^, and *Jnk1*^*Δhepa*^/*2*^−/−^ mice 28 days after BDL. Sham-operated mice were used as controls (n = 5–6 animals per genotype). (*J*) CK19-positive cells were quantified as an area percentage of liver sections (n = 5–6 animals per genotype; scale bars, 100 μm). Data are shown as means ± SEM from 5 to 6 mice per group (*n*, non-significant; *∗∗P* < .01, *∗∗∗P* < .001, *∗∗∗∗P* < .0001; 1-way ANOVA with Bonferroni’s multiple comparisons test).

Recently, we demonstrated that *Jnk1* and *Jnk2* cooperate in hepatocytes to protect against cholestatic liver injury.^15^ Kluwe et al reported that *Jnk2* deletion following BDL increases fibrosis in contrast to *Jnk1*^*-/-*^ animals.^14^ To test whether *Jnk2* in nonhepatocytes confers additional protection after BDL, we generated mice with hepatocyte-specific deletion of *Jnk1* and global ablation of *Jnk2* (*Jnk1*^*Δhepa*^*/2*^*−/−*^). As we previously reported, long-term inactivation of global *Jnk2* in *Jnk2*^*-/-*^ or *Jnk1*^*Δhepa*^*/2*^*-/-*^ mice did not trigger liver injury compared with WT controls up to 6 months of age. However, at 52 weeks of age, *Jnk1*^*Δhepa*^*/2*^*-/-*^ but not *Jnk2*^*-/-*^ mice developed cyst formation and increased ductular reaction associated with a mild increase in typical serum parameters of liver damage, including AST, alanine aminotransferase (ALT), and alkaline phosphatase (AP).^17^^,^^18^ Kaplan-Meier analysis demonstrated that increased hepatic damage in the *Jnk1*^*Δhepa*^*/2*^*-/-*^ mice had no significant impact on survival compared with *Jnk2*^*-/-*^ or WT mice (Figure 3*A*). Histopathologic examination of extrahepatic organs such as heart, kidney, lung, spleen, and pancreas showed no evidence of damage or pathology in these organs (data not shown). Additionally, serum levels of pancreatic amylase were comparable between the studied genotypes, indicating that the pancreatic activity was neither affected in response to *Jnk2* deletion in both *Jnk2*^*-/-*^ and *Jnk1*^*Δhepa*^*/2*^*-/-*^ mice (Figure 3*B*).

Next, these mice were subjected to BDL and were compared with *Jnk2*^*-/-*^ or mice with hepatocyte-specific deletion of *Jnk1 and Jnk2* (*Jnk1*^*Δhepa*^*/2*^*Δhepa*^). WT floxed mice were included as controls. The presence of yellowish dots was macroscopically obvious on BDL-ligated WT and *Jnk2*^*-/-*^ livers, whereas *Jnk1*^*Δhepa*^*/2*^*−/−*^ livers exhibited large foci on their surface, suggestive of enhanced liver damage (Figure 3*C*). Moreover, BDL triggered necrotic areas in the hepatic parenchyma of WT and *Jnk2*^*-/-*^ animals (Figure 3*D–E*). In contrast, dramatically increased necrotic foci were visible in the liver parenchyma of *Jnk1*^*Δhepa*^*/2*^*−/−*^ animals (Figure 3*D–E*). They were also larger compared with *Jnk1*^*Δhepa*^*/2*^*Δhepa*^ livers, 28 days after BDL (Figure 3*G–H*). Consistently, serum AST levels increased approximately 10-fold and 12-fold in WT and *Jnk2*^*-/-*^ mice, respectively, compared with sham controls. However, AST levels in BDL-treated *Jnk1*^*Δhepa*^*/2*^*−/−*^ mice increased significantly compared with BDL-treated WT and *Jnk2*^*-/-*^ animals (Figure 3*F*).

Concomitantly, cytokeratin-19 (CK-19) staining showed that BDL triggered increased ductular proliferation, which was most pronounced in BDL-treated *Jnk1*^*Δhepa*^*/2*^*−/−*^ mice (Figure 3*I–J*). Altogether, these data depict a crucial protective effect of *Jnk1* in hepatocytes and a more global role of *Jnk2* during experimental cholestatic liver disease.

### BDL-induced Immune Cell Recruitment is Increased in *Jnk1*^*Δhepa*^*/2*^*−/−*^ Mice

**Figure 4:**
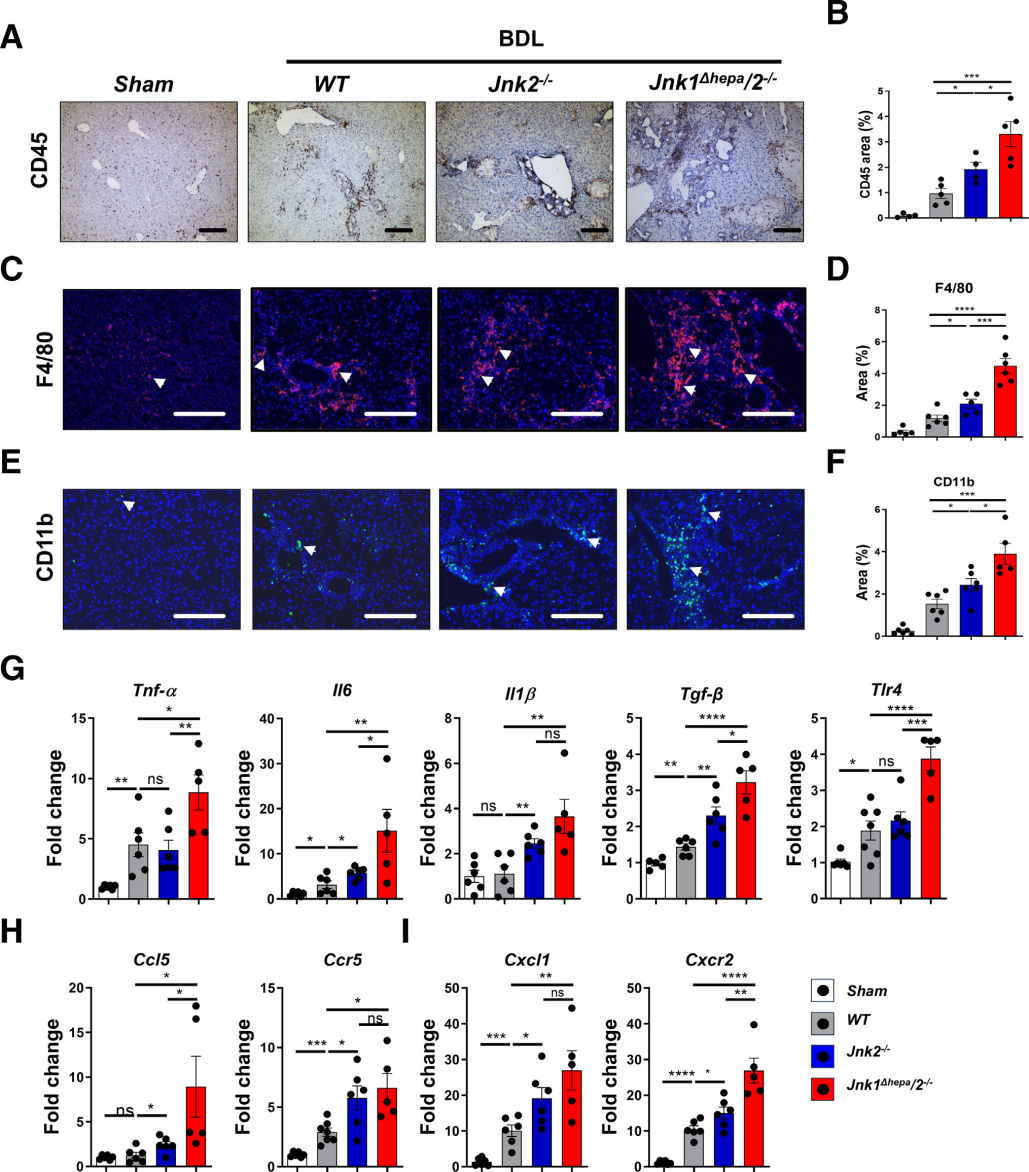
**BDL-induced immune cell recruitment is increased in *Jnk1*^*Δhepa*^*/2*^*−/−*^ mice.***Jnk*^*f/f*^ (WT), *Jnk2*^−/−^ and *Jnk1*^*Δhepa*^/*2*^−/−^ mice were subjected to BDL for 28 days. Sham-operated mice are used as controls (n = 5–6 per genotype). (*A*) Representative IHC of CD45 staining in the liver sections (Scale bar, 100 μm). (*B*) CD45-positive areas were quantified. (*C–F*) Representative IF and quantification of F4/80 and CD11b staining in liver sections of the same mice; sham-operated mice are used as controls (n = 5–6 animals per genotype). Positive cells were quantified as an area percentage of the liver sections (Scale bars, 100 μm). (*G–I*) Hepatic expressions of *Tnf-α*, *Il6, Il1β, Tgf-β*, *Tlr4, Ccl5, Ccr2, Cxcl1* and *Cxcr2* mRNA was determined by qRT-PCR and normalized to *Gapdh* mRNA level. Data are shown as means ± SEM (*ns*, non-significant; *∗P* < .05, *∗∗P* < .01, *∗∗∗P* < .001, *∗∗∗∗P* < .0001; 1-way ANOVA with Bonferroni’s multiple comparisons test).

Immune cell infiltration and inflammatory mediators (eg, cytokines and chemokines) have a major impact on directing hepatic cholestasis. Following BDL, the number of infiltrating immune cells increased in WT compared with sham-operated mice as shown by CD45 staining (Figure 4*A–B*). Immune cell activation significantly increased in *Jnk2*^*-/-*^ and *Jnk1*^*Δhepa*^*/2*^*−/−*^ livers after BDL (Figure 4*A–B*). A detailed analysis revealed that this effect is mainly mediated by hepatic macrophages as shown by CD11b and F4/80 staining (Figure 4*C–F*). These results suggest that JNKs play cell-specific roles during cholestasis. While synergistic protective mechanisms of JNK1 and JNK2 are important in hepatocytes, JNK2 might modulate inflammation and immune cell activation in nonhepatocytes.

Next, we analyzed which immune mediators direct immune cell activation after BDL. *Jnk2*^*-/-*^ or *Jnk1*^*Δhepa*^*/2*^*−/−*^ mice triggered significantly higher mRNA expression of *Il6, Il1β, and Tgf-β* compared with controls. In contrast, both *tumor necrosis factor-alpha* (*Tnf-α*) and toll-like receptor 4 (*Tlr4)* were only significantly increased in *Jnk1*^*Δhepa*^*/2*^*−/−*^ but not *Jnk2*^*-/-*^ animals when compared with BDL-treated WTs (Figure 4*G*). Consistent with increased F4/80/CD11b expression, the Ccl5-Ccr5 axis, mediating macrophage recruitment and activation,^19^ was significantly upregulated in *Jnk1*^*Δhepa*^*/2*^*−/−*^ and *Jnk2*^*-/-*^ compared with WT livers (Figure 4*H*). Besides, CXC motif chemokine receptor 2 (CXCR2) triggering the CXC motif chemokine ligand 1 (CXCL1)-CXCR2 axis, involved in neutrophil recruitment, was significantly upregulated when JNK2 was completely deleted (Figure 4*I*). Importantly, these effects were more pronounced in *Jnk1*^*Δhepa*^*/2*^*−/−*^ livers, suggesting that *Jnk2* expression in nonhepatocytes might be involved in modulating specific subsets of immune mediators controlling the activation/recruitment of macrophages and neutrophils during chronic cholestasis.

### JNK Signaling Is Involved in Cholestatic Liver Disease-derived Fibrogenesis

**Figure 5:**
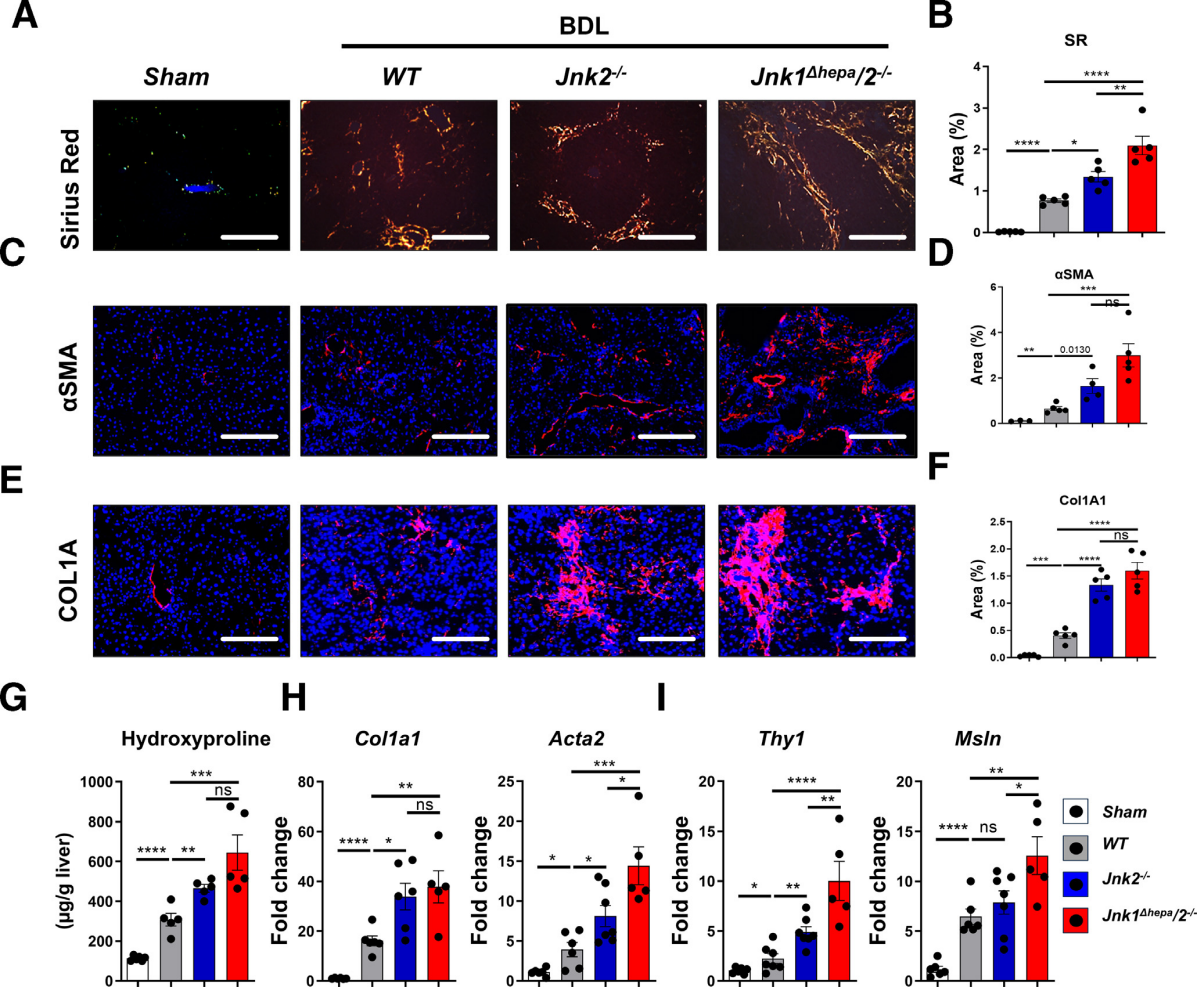
**Chronic BDL induces increased fibrogenesis in *Jnk1*^*Δhepa*^*/2*^*−/−*^ livers.***Jnk*^*f/f*^ (WT), *Jnk2*^−/−^, and *Jnk1*^*Δhepa*^/*2*^−/−^ mice were subjected to BDL for 28 days. Sham-operated mice are used as controls (n = 5–6 per genotype). (*A–B*) Representative imaging and quantification of SR staining in sections of liver tissue harvested from the same mice (Scale bars, 100 μm). (*C–F*) Representative IF and quantification of αSMA and COL1A staining in liver sections of the same mice, sham-operated mice are used as controls (n = 5–6 animals per genotype). Positive cells were quantified as an area percentage of the liver sections (n = 5–6 animals per genotype; scale bars, 100 μm). (*G*) Hydroxyproline assay was performed in liver samples from the same mice as indicated. (*H–I*) Hepatic *Col1a1, Acta2, Thy1, and Msln* mRNA expression was determined by qRT-PCR and normalized to *Gapdh* mRNA level. Data are shown as means ± SEM from 5 to 6 mice per group (*n*, non-significant; *∗P* < .05, *∗∗P* < .01, *∗∗∗P* < .001, *∗∗∗∗P* < .0001; 1-way ANOVA with Bonferroni’s multiple comparisons test).

Chronic cholestasis triggers fibrosis progression via extracellular matrix (ECM) deposition. BDL in WT livers triggered fibrous tissue and septum formation surrounding the portal areas demonstrated by SR staining (Figure 5*A–B*). Concomitantly, SR quantification revealed increased SR-positive areas in *Jnk2*^*-/-*^ and the highest values in *Jnk1*^*Δhepa*^*/2*^*−/−*^ livers (Figure 5*A–B*). Interestingly, alpha-smooth muscle actin (αSMA) protein expression (indicative of HSCs activation) analysis showed a significant increase in BDL-treated *Jnk2*^*-/-*^ vs WT animals (Figure 5*C–D*). Despite more severe liver injury in *Jnk1*^*Δhepa*^*/2*^*−/−*^ mice, αSMA-positive areas were not increased compared with *Jnk2*^*-/-*^ animals but were significantly higher compared with WT livers (Figure 5*C–D*). These results were further validated by Col IA immunofluorescence (IF) (Figure 5*E–F*) and significantly higher hydroxyproline content in *Jnk2*^*-/-*^ and *Jnk1*^*Δhepa*^*/2*^*−/−*^ mice compared with controls (Figure 5*G*). These findings were confirmed by measuring *Col1a1, Acta2, Thy1, and* mesothelin ***(****Msln)* mRNA expression to investigate myofibroblast activation and ECM deposition (Figure 5*H–I*). Altogether, these results show that chronic BDL induces stronger liver injury and increased fibrogenesis in *Jnk2*^*−/−*^, which is more pronounced in *Jnk1*^*Δhepa*^*/2*^*−/−*^ livers.

### *Jnk1-* and *Jnk2*-specific Gene Regulation in Hepatocytes and Nonparenchymal Liver Cells After BDL

**Figure 6:**
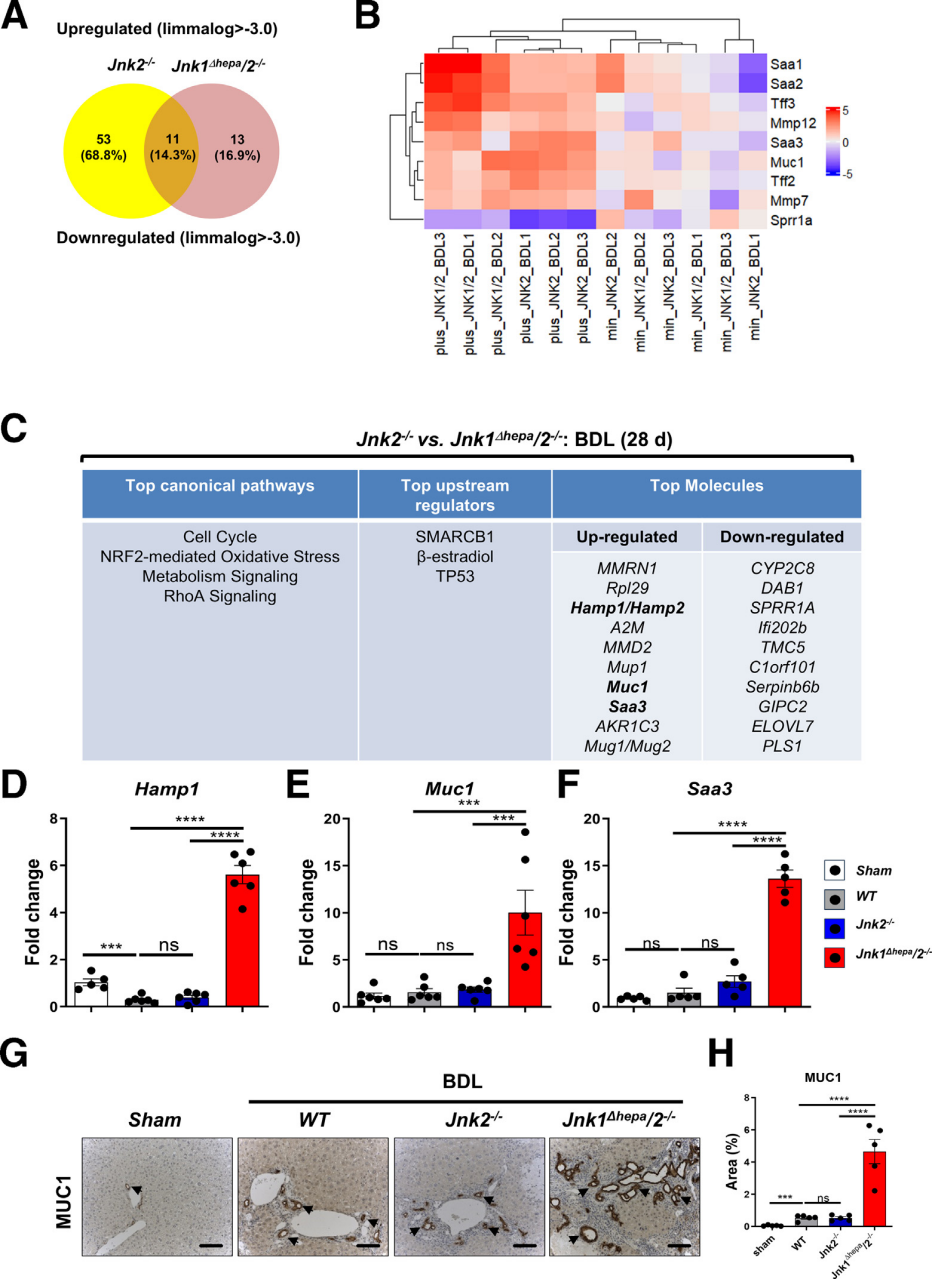
**Loss of*Jnk2*in NPCs plus*Jnk1*/*2* in hepatocytes triggered oxidative stress burst, overexpression of acute phase response, and metabolic signaling.** Microarray was performed from *Jnk2*^*-/-*^ and *Jnk1*^*Δhepa*^*/2*^*-/-*^ livers 28 days after BDL. Log2 expression values of the individual mice were divided by the mean of the sham-operated mice. Log ratios were saved in a .txt file and analyzed with the Multiple Experiment Viewer. (*A*) The percentage of upregulated and downregulated genes. (*B*) Heat map of the top up- and down-regulated genes (*red*: upregulated; *green*: downregulated, n = 3, FC, –5.0 to 5.0). (*C*) Ingenuity Pathway Analysis was performed in the same liver samples. The top canonical pathways (*left panel*), the top upstream regulators (*middle panel*), and the top up-and down-regulated genes (*right panel*) are shown. (*D–F*) Hepatic *Hamp1, Muc1, and Saa3* mRNA expression was determined by qRT-PCR and normalized to *Gapdh* mRNA. (*G–H*) Representative IHC and quantification of MUC1 staining in the liver sections (Scale bar, 100 μm). Data are shown as means ± SEM from 5 to 6 mice per group (*n*, non-significant; *∗P* < .05, *∗∗P* < .01, *∗∗∗P* < .001, *∗∗∗∗P* < .0001; 1-way ANOVA with Bonferroni’s multiple comparisons test).

To dissect pathways between *Jnk1*^*Δhepa*^*/2*^*−/−*^ vs *Jnk2*^*−/−*^ livers, we performed Affymetrix GeneChip microarray analyses, after BDL, revealing significant differences (−5.0<fold change [FC]>5.0) in gene expression (Figure 6*A–B*). First, hierarchical clustering of genes commonly up- or down-regulated in both *Jnk2*^*−/−*^ and *Jnk1*^*Δhepa*^*/2*^*−/−*^ livers were performed (Figure 6*C*). Significantly increased signaling pathways in both mouse livers included genes involved in cell cycle, oxidative stress homeostasis (Nrf2 pathway), metabolism, and RhoA signaling. Additionally, transcript levels of IL6-dependent target genes, including *Saa3* and *Hamp1*, were upregulated in both genotypes (Figure 6*C*). Specifically, *Jnk1*^*Δhepa*^*/2*^*−/−*^ livers displayed increased transcripts of genes related to mucosal and epithelium protection (Mucin), whereas the present study identified downregulation of the small proline-rich repeat protein (SPRR) gene family members SPRR1A, a stress-inducible protein regulated by the Il6/gp130 signaling pathway (Figure 6*C*).

To validate these findings, mRNA expression was analyzed in *Jnk2*^*−/−*^ and *Jnk1*^*Δhepa*^*/2*^*−/−*^ livers, 28 days after BDL, revealing significantly increased hepatic expression of *Hamp1, Muc1, and Saa3* (Figure 6*D–F*), which was confirmed by immunohistochemical (IHC) staining for MUC1 reflecting the stronger ductular reaction seen in *Jnk1*^*Δhepa*^*/2*^*−/−*^ mice (Figure 6*G*).

**Figure 7:**
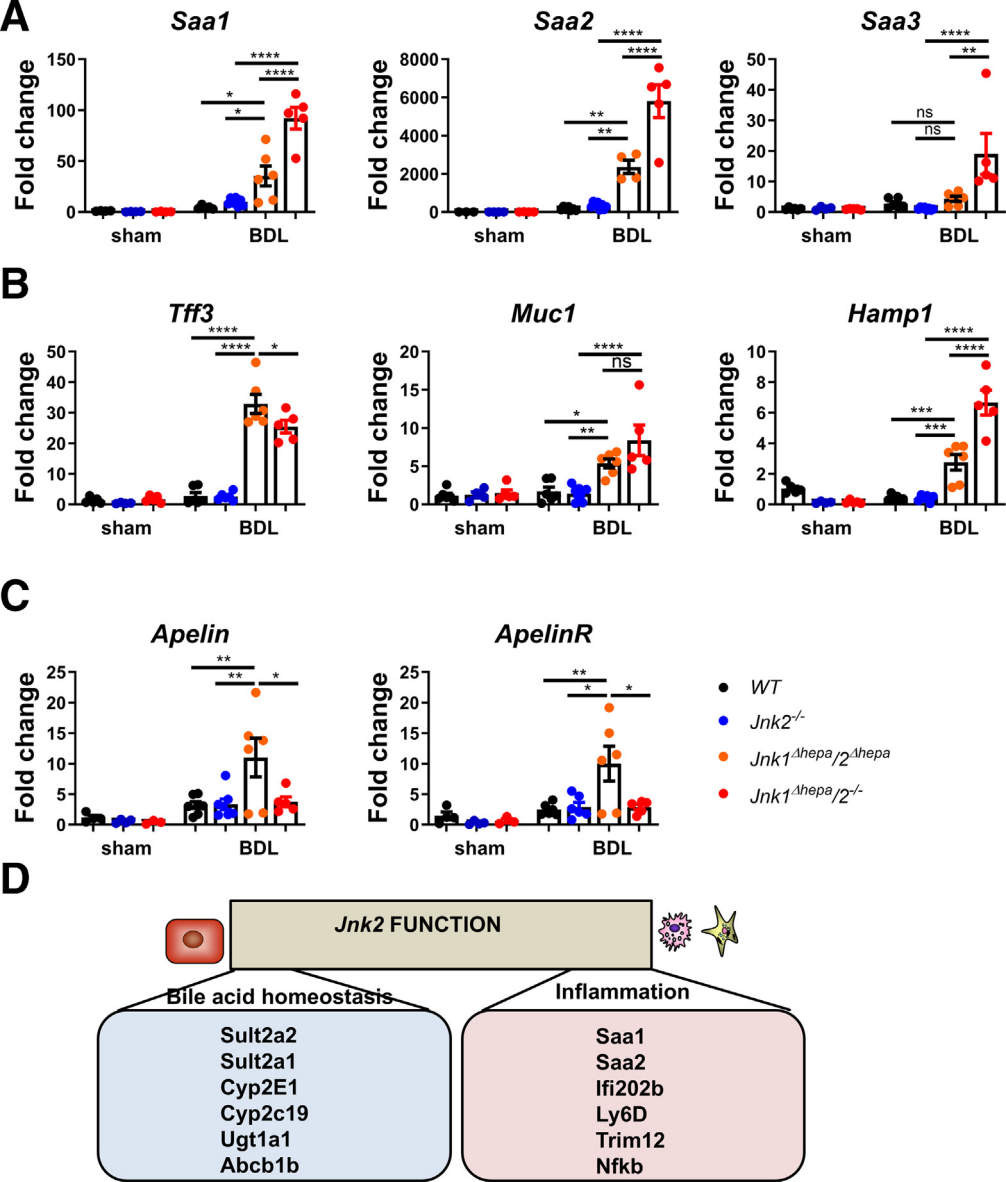
***Jnk2* specific gene regulation in hepatocytes and nonparenchymal liver cells in experimental cholestasis.***Jnk*^*f/f*^ (WT), *Jnk2*^−/−^, *Jnk1*^*Δhepa*^/*2*^*Δhepa*^, and *Jnk1*^*Δhepa*^/*2*^−/−^ mice were subjected to BDL for 28 days. Sham-operated mice are used as controls (n = 5–6 per genotype). (*A–C*) Hepatic *Saa1, Saa2, Saa3, Tff3, Muc1, Hamp1, Apelin, and ApelinR* mRNA expression in total liver tissue harvested from the same mice determined by qRT-PCR and normalized to *Gapdh* RNA level (n = 5–6 animals per genotype for each time point). Data are shown as means ± SEM (*ns*, non-significant, *∗P* < .05, *∗∗P* < .01, *∗∗∗P* < .001, *∗∗∗∗P* < .0001; 1-way ANOVA with Bonferroni’s multiple comparisons test). (*D*) A schematic diagram shows the differentially expressed genes via *Jnk2* in hepatocytes and NPCs.

*Jnk1*^*Δhepa*^*/2*^*-/-*^ animals had overlapping results compared with *Jnk1*^*Δhepa*^*/2*^*Δhepa*^ mice.^15^ To validate the contribution of *Jnk* expression in hepatocytes vs nonhepatocytes, we compared the expression of prominently controlled genes on the mRNA level after BDL, using these different animals. Acute phase response genes were increased in WT livers (*Saa1-3*) (Figure 7*A*). However, they further increased in *Jnk2*^*-/-*^ and *Jnk1*^*Δhepa*^*/2*^*Δhepa*^ but were highest in *Jnk1*^*Δhepa*^/*2*^*-/-*^ livers. Hence, the regulation of acute-phase genes correlated with the degree of loss of Jnk-dependent protection and disease progression during cholestatic liver injury.

In contrast, *Muc1, Hamp1,* and *Tff3* were only upregulated when both *Jnk1* and *Jnk2* were deleted at least in hepatocytes, with a prominent increase of *Muc1 and Hamp1* but not *Tff3* in *Jnk1*^*Δhepa*^/*2*^*-/-*^ compared with *Jnk1*^*Δhepa*^*/2*^*Δhepa*^ livers (Figure 7*B*). *Apelin* and *ApelinR* expressions were significantly upregulated in *Jnk1*^*Δhepa*^*/2*^*Δhepa*^ livers after BDL, suggesting that a JNK2-dependent negative feedback loop in NPCs is present (Figure 7*C*).

Next, we studied the specific role of *Jnk* genes in parenchymal and nonhepatocytes during experimental cholestasis. Here, data from our previous work^15^ and the current microarray analysis were compared using Limma log2 (FC). Differences in gene regulation between both conditions were selected and depicted. Specifically, we were interested in genes that were differentially regulated after *Jnk1/2* deletion in hepatocytes vs *Jnk2* global mutants (Figure 7*D*). Interestingly, we found a dual role for *Jnk2* during cholestasis. *Jnk2-*regulated genes in hepatocytes are involved in energy and BA metabolism (*Sult2a2, Cyp2e1, Cyp2c19, Ugt1a1* and *Abcb1b*), whereas in nonhepatocytes *Jnk2* controls inflammatory processes (*Saa1/2, Ifi202b, Ly6DTrim12,* and *Nfkb*) (Figure 7*D*), suggesting that *Jnk2* in nonhepatocytes modulates the proinflammatory response during cholestasis.

### *Jnk2* in BM-derived Immune Cells Does Not Mediate the Effect After BDL

**Figure 8:**
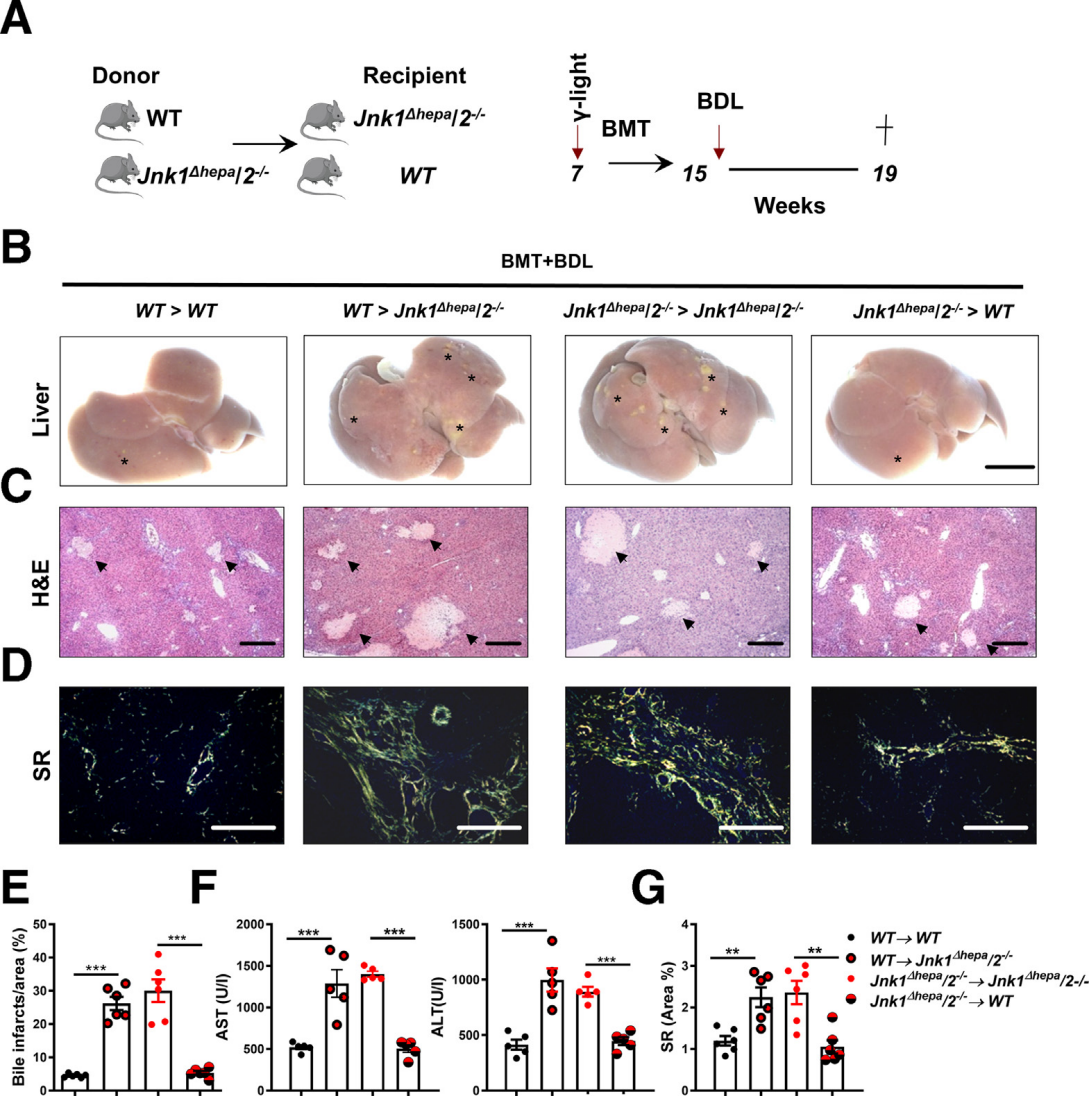
**BMT excluded the role of *Jnk2* in BM-derived cells in *Jnk1*^*Δhepa*^/*2*^−/−^ phenotype after BDL.** (*A*) The cartoon visualizes the experimental design of BMT followed by BDL. BM from *Jnk1*^*Δhepa*^*/2*^*−/−*^ and WT mice were transferred into *Jnk1*^*Δhepa*^*/2*^*−/−*^ isogeneic recipients (*n* = 6–7 mice per group) after ablative γ-irradiation. Two months after BMT, the mice underwent BDL for 28 days. (*B*) Macroscopic appearance of chimeric WT and *Jnk1*^*Δhepa*^/*2*^−/−^ livers transplanted with WT and *Jnk1*^*Δhepa*^/*2*^−/−^ BM cells, followed by BDL for 28 days. (∗) indicates yellow dots on the liver surface. (*C*) Representative H&E-stained liver sections from these mice (→). Bile infarcts corresponding to the yellow dots observed on the liver surface. (*D*) Imaging of SR staining in sections of liver tissue (Scale bars, 100 μm). (*E*) Quantification of bile infarcts in the chimeric mice after BDL. (*F*) Serum AST and ALT. (*G*) Quantification of SR-positive area in the liver sections from the same mice. Data are shown as means ± SEM from 5 to 6 mice per group (*∗∗P* < .01, *∗∗∗P* < .001; 1-way ANOVA with Bonferroni’s multiple comparisons test).

Next, we investigated which nonhepatocytes mediate this effect and performed bone marrow transplantation (BMT) experiments to assess the role of BM-derived cells. *WT* and *Jnk1*^*Δhepa*^/*2*^*-/-*^ mice were lethally irradiated and reconstituted with either *Jnk1*^*Δhepa*^/*2*^*-/-*^ or *WT* BM, followed by BDL for 28 days (Figure 8*A*). Chimerism in the liver was confirmed by polymerase chain reaction (PCR) demonstrating successful BM reconstitution (not shown).

Macroscopic and microscopic evaluation 28 days after BDL of BMT-treated animals revealed that livers with combined *Jnk1* and *Jnk2* deletion and reconstituted either with WT (WT→*Jnk1*^*Δhepa*^/*2*^*-/-*^) or *Jnk1*^*Δhepa*^/*2*^*-/-*^ BM (*Jnk1*^*Δhepa*^/*2*^*-/-*^→*Jnk1*^*Δhepa*^/*2*^*-/-*^) displayed larger bile infarcts and enhanced liver damage compared with reconstituted WT livers (Figure 8*B–C and E*), suggesting that combined deletion of *Jnk1* and *Jnk2* in hepatocytes is essential to determine the phenotype of liver injury. Surprisingly, reconstitution of *Jnk1*^*Δhepa*^/*2*^*-/-*^ mice with WT or *Jnk1*^*Δhepa*^/*2*^*-/-*^ BM also did not significantly affect the increased number of necrotic foci compared with reconstituted WT mice after BDL (Figure 8*C and E*). These data were supported by serum AST and ALT levels (Figure 8*F*). Consequently, fibrosis assessment using SR staining was not changed upon BM reconstitution in *Jnk1*^*Δhepa*^/*2*^*-/-*^ livers (Figure 8*D and G*). These results suggest that JNK2 in NPC and not BM-derived immune cells are crucial in controlling the inflammatory response and immune cell recruitment during chronic cholestasis.

### JNK Deficiency Aggravates Hepatic BA Accumulation During Cholestasis

**Figure 9:**
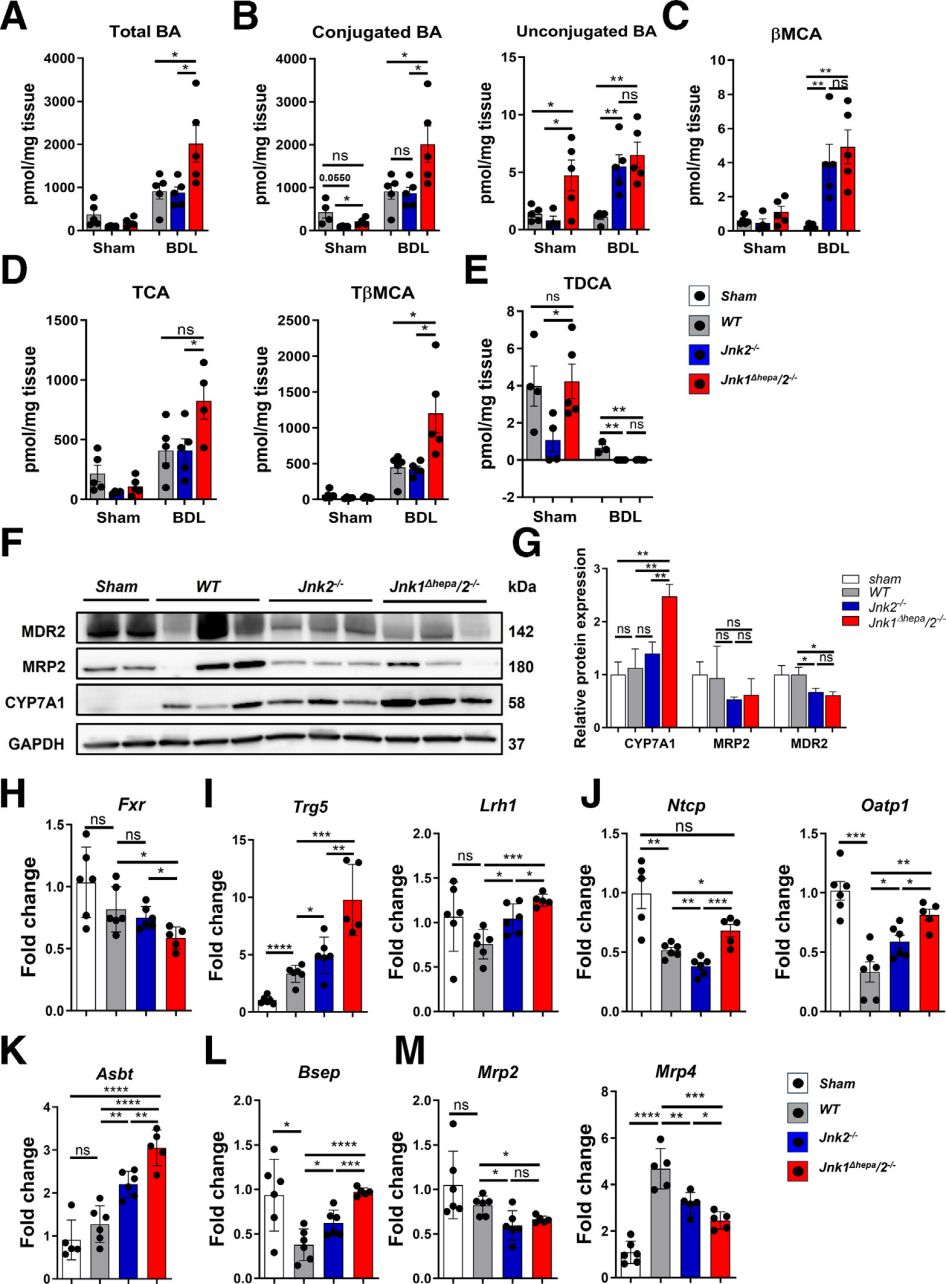
**Hepatic BA content and expression of genes involved in BA metabolism in *Jnk2*^*f/f*^*(*WT*), Jnk2*^−/−^ and *Jnk1*^*Δhepa*^*/2*^*−/−*^ mice after BDL.** (*A–B*) Total, primary, secondary, and conjugated BA concentrations were measured in livers after BDL or sham surgery (*Jnk2*^*f/f*^ (WT), *Jnk2*^−/−^ and *Jnk1*^*Δhepa*^/*2*^−/−^; n = 5). (*C–E*) Hepatic βMCA, TCA, TβMCA, and TDCA were determined in the same livers (n = 5). (*F*) Immunoblot analysis of liver extracts for CYP7A1, MRP2, and MDR2, using GAPDH as a loading control. (*G*) The density of the CYP7A1, MRP2, and MDR2 bands were quantified and compared with the GAPDH intensity accordingly. (*H–M*) Hepatic *Fxr, Trg5, Lrh1, Ntcp, Oatp1, Asbt, Bsep, Mrp2,* and *Mrp4* mRNA expression was determined by qRT-PCR. Data are shown as means ± SEM from 5 to 6 mice per group (*n*, non-significant, *∗P* < .05, *∗∗P* < .01, *∗∗∗P* < .001, *∗∗∗∗P* < .0001; 1-way ANOVA with Bonferroni’s multiple comparisons test).

As BDL triggers hepatic BA accumulation, we next assessed whether single or combined loss of *Jnk2* activity has an impact on BAs after BDL in both *Jnk2*^*-/-*^ and *Jnk1*^*Δhepa*^/*2*^*-/-*^ compared with WT livers. In sham-operated controls, the livers of both *Jnk2*^*-/-*^ and *Jnk1*^*Δhepa*^/*2*^*-/-*^ mice exhibited lower content of total BAs when compared with WT, but the difference did not reach significance between the studied groups. (Figure 9*A*). As expected, BDL triggered the accumulation of total BAs compared with sham mice. WT and *Jnk2*^*-/-*^ livers reached comparable levels, whereas in *Jnk1*^*Δhepa*^/*2*^*-/-*^ liver BAs were significantly increased (Figure 9*A*), which was primarily due to an increase of conjugated BAs in the BDL-treated *Jnk1*^*Δhepa*^/*2*^*-/-*^ livers compared with WT and *Jnk2*^*-/-*^ livers (Figure 9*B*). Interestingly, the unconjugated BA content was significantly higher in *Jnk1*^*Δhepa*^/*2*^*-/-*^ liver of sham-operated mice compared with WT or *Jnk2*^*-/-*^ livers, whereas BDL led to an increase of unconjugated BAs in *Jnk2*^*-/-*^ but not *Jnk1*^*Δhepa*^/*2*^*-/-*^ livers (Figure 9*B*).

Detailed BA profiling revealed a significant increase in overall β-muricholic acid (βMCA) in both *Jnk2*^*-/-*^ and *Jnk1*^*Δhepa*^/*2*^*-/-*^ liver compared with WT livers (Figure 9*C*), whereas taurocholic acid (TCA) and β-tauromuricholic acid (TβMCA) species were exclusively significantly increased in *Jnk1*^*Δhepa*^/*2*^*-/-*^ liver compared with WT and *Jnk2*^*-/-*^ livers after BDL (Figure 9*D*). Inversely, taurodeoxycholic acid (TDCA) was significantly reduced after BDL but was nondetectable in *Jnk2*^*-/-*^ and *Jnk1*^*Δhepa*^/*2*^*-/-*^ livers (Figure 9*E*). To investigate the cause of elevated hepatic BA levels in *Jnk1*^*Δhepa*^/*2*^*-/-*^ mice, we assessed the protein expression of the BA synthesis pathways.

Concomitant with the accumulation of TβMCA, a known farnesoid X receptor (FXR) antagonist, we found downregulation of *Fxr* mRNA expression in *Jnk1*^*Δhepa*^/*2*^*-/-*^ livers compared with *Jnk2*^*-/-*^ and WT animals after BDL (Figure 9*H*). Although *Tgr5* and *Lrh1* expressions were significantly upregulated in both genotypes after BDL (Figure 9*I*). Interestingly, protein expression of CYP7A1, the rate-limiting enzyme of BA synthesis, was increased in *Jnk2*^*-/-*^ with a prominent increase in *Jnk1*^*Δhepa*^/*2*^*-/-*^ livers compared with WT livers after BDL (Figure 9*F–G*), These findings suggest that hepatocytic JNK1 and JNK2 deficiencies trigger hepatic BA overload by enhancing BA synthesis and disrupting feedback regulation.

### Disruption of the Hepatic Biliary Transport System in *Jnk1*^*Δhepa*^*/2*^*−/−*^ Mice

Inflammation has been shown to control hepatobiliary transporter expression,^5^ hence we analyzed the impact of *Jnk* loss on their expression. After BDL, the expressions of BA uptake transporters *Ntcp* and *Oatp1* were reduced in all genotypes compared with sham-operated mice. However, after BDL *Oatp1* expression was highest when *Jnk2* was deleted in NPCs (*Jnk2*^*-/-*^ and *Jnk1*^*Δhepa*^/*2*^*-/-*^ compared with WT livers), whereas *Ntcp* increased specifically in *Jnk1*^*Δhepa*^/*2*^*-/-*^ compared with WT livers (Figure 9*J*).

Likewise, the hepatic apical sodium-dependent BA transporter (ASBT) was significantly increased in *Jnk2*^*-/-*^ and *Jnk1*^*Δhepa*^/*2*^*-/-*^ compared with WT livers (Figure 9*K*). On the other hand, *Bsep* expression was downregulated after BDL but significantly increased in *Jnk2*^*-/-*^ with a prominent increase in *Jnk1*^*Δhepa*^/*2*^*-/-*^ livers compared with WT livers (Figure 9*L*). At the same time, the expression of MRP transporter family members (*Mrp2* and *Mrp4*) was significantly downregulated in both *Jnk2*-deficient genotypes (Figure 9*M*). These data were confirmed by Western blot analysis showing MDR2 and MRP2 protein levels were lower in *Jnk2*^*-/-*^ and *Jnk1*^*Δhepa*^/*2*^*-/-*^ compared to WT livers after BDL (Figure 9*F–G*).

Altogether, these data suggest that JNKs trigger hepatic BA accumulation via disruption of BA synthesis and transporter expression.

### Expression of Human BA Transporters Is Downregulated in Patients With Cholestatic Liver Disease

**Figure 10:**
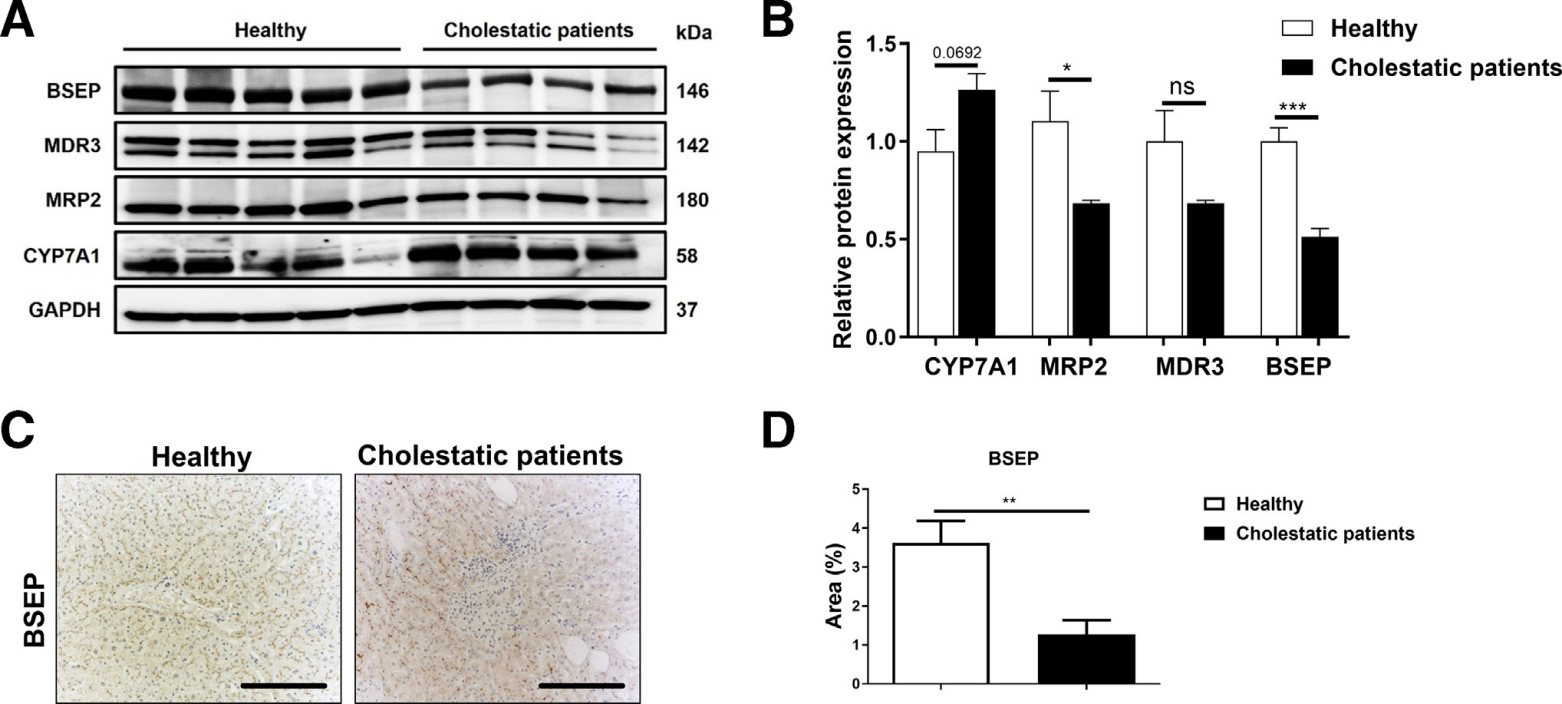
**Dysregulation of BA synthesis and transport in patients with cholestasis.** (*A*) immunoblot analysis of liver extracts prepared from cholestatic patients (n = 4) and healthy individuals (n = 5) were examined for CYP7A1, MRP2, MDR3, and BSEP, using GAPDH as loading control. (*B*) The density of the CYP7A1, MRP2, MDR3, and BSEP bands was quantified and compared with the GAPDH intensity accordingly. (*D*) Representative IHC of BSEP staining in liver sections from cholestatic patients and healthy individuals. (*D*) The BSEP-positive area was quantified and blotted. Data are shown as means ± SEM (*n*, non-significant, *∗P* < .05, *∗∗P* < .01; unpaired, 2-tailed Student *t*-test).

To investigate the translational relevance of our findings in mouse models, we extended our previous study on JNK activation in human cholestatic liver disease by analyzing the expression of key proteins involved in BA synthesis and transport in the same patient cohort.^15^ Specifically, we assessed protein expression of the BA synthesis enzyme CYP7A1 and the BA transporters proteins, BSEP, MRP2, and MDR3 (encoded by *Mdr2* in mice) in livers from cholestatic patients by Western blot. CYP7A1 protein expression was upregulated in cholestatic patients compared with healthy controls (Figure 10*A–B*). Oppositely, BSEP, MRP2, and MRD3 protein expression were downregulated in cholestatic patients compared with healthy controls (Figure 10*A–B*). Furthermore, immunostaining in liver sections for BSEP in cholestatic patients confirmed its reduced expression (Figure 10*C–D*).

### IL6 Regulates Hepatic Transporters Via a JNK2-dependent Mechanism

**Figure 11:**
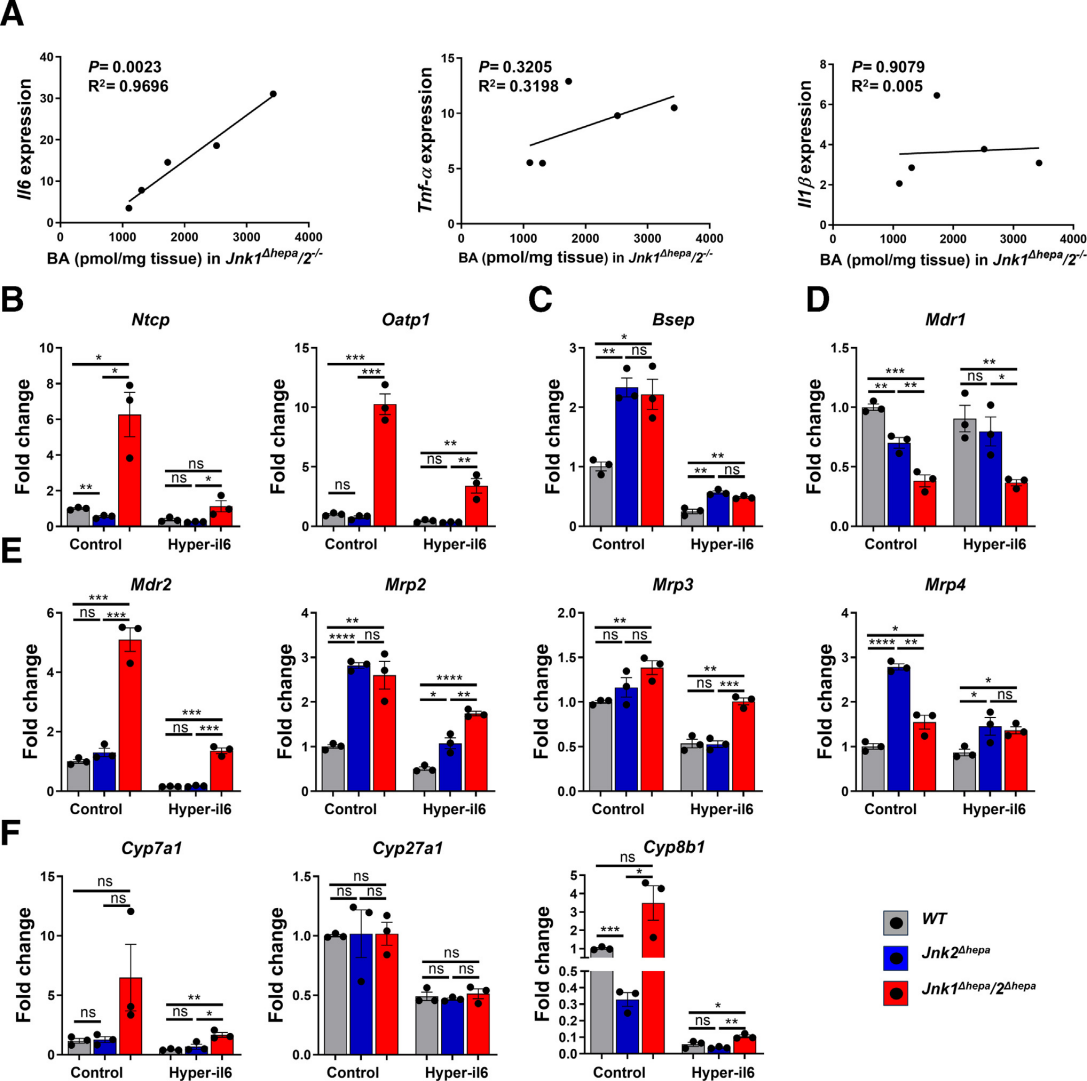
**IL6 regulates hepatic transporters via a JNK2-dependent mechanism.** (*A*) Linear regression analysis of hepatic mRNA expression of *Il6, Tnf-α,* and *Il1β* vs total hepatic BA composition in *Jnk1*^*Δhepa*^*/2*^*−/−*^ livers after BDL. (*B–F*) Primary hepatocytes were isolated from *Jnk*^*f/f*^ (WT), *Jnk2-*, and *Jnk1*/*2-*deficient mice. The cells were treated with or without 50 ng/mL H-IL6 for 24 hours. mRNA expression of *Ntcp, Oatp1, Bsep, Mdr1, Mdr2, Mrp2* and *Mrp3, Mrp4, Cyp7a1, Cyp27a, and Cyp8b1* mRNA in *Jnk*^*f/f*^ (WT), *Jnk2-*, and *Jnk1*/*2-*deficient hepatocytes was determined by qRT-PCR. Data are shown as means ± SEM from 3 independent experiments (*n*, non-significant, *∗P* < .05, *∗∗P* < .01, *∗∗∗P* < .001, *∗∗∗∗P* < .0001; 1-way ANOVA with Bonferroni’s multiple comparisons test).

To functionally investigate how BA accumulation is aggravated in *Jnk1*^*Δhepa*^/*2*^*-/-*^ livers, we hypothesized that higher cytokine expression, specifically of IL6, is involved in mediating the decrease in BA transporter expression.^20^, ^21^, ^22^ Consistently, hepatic *Il6* mRNA expression in *Jnk1*^*Δhepa*^/*2*^*-/-*^ mice correlated significantly with total bile composition but not with *Tnf-α* or *Il1β* expression (Figure 11*A*). We stimulated primary hepatocytes from *Jnk2* or combined *Jnk1*/*Jnk2* knockouts with Hyper-IL6 (H-IL6).

The basal expression of the BA uptake transporters, *Ntcp* and *Oatp1*, was strongly enhanced in *Jnk1/2-*deficient hepatocytes compared with WT and *Jnk2-*deficient hepatocytes (Figure 11*B*). Twenty-four-hour H-IL6 stimulation downregulated both BA uptake transporters in WT, *Jnk2*-, and *Jnk1/2*-deficient hepatocytes compared with untreated controls (Figure 11*B*).

Basal expression of the BA efflux transporters *Bsep* was lower in WT compared with both *Jnk2* KO groups, and H-IL6 reduced its expression in all 3 groups (Figure 11*C*). Loss of *Jnk2* or *Jnk1/2* in hepatocytes significantly increased the expression of *Mdr2, Mrp2, Mrp3,* and *Mrp4* in nontreated hepatocytes*,* whereas H-IL6 stimulation reduced their expression in all 3 groups (Figure 11*E*). In contrast, *Mdr1* expression was suppressed in *Jnk2-* and *Jnk1/2*-deficient hepatocytes (Figure 11*D*). Along with reduced BA transporter expression, H-IL6 significantly reduced the expression of genes involved in BA synthesis (*Cyp7a1, Cyp27a1,* and *Cyp8b1)* in WT, *Jnk2*- and *Jnk1/2*-deficient hepatocytes compared with untreated groups (Figure 11*F*).

In summary, IL6 directly controls BA transporter expression; hence, the increased inflammatory response in JNK2 knockout mice might directly contribute to the dysregulation of BA homeostasis.

## Discussion

Hepatic cholestasis arises from the accumulation of toxic bile salts in the liver due to impaired BA flow. The balance between synthesis, uptake, and export of BA is crucial to maintain enterohepatic BA circulation and facilitate successful BA detoxification. Disruption of the BA homeostasis is associated with cholestasis in humans and mice.^23^ Previously, JNK activation was reported in human and murine cholestasis and is involved in the regulation of BA metabolism.^24^, ^25^, ^26^, ^27^ However, the impact of JNK isoforms and their cell-specific functions in hepatocytes vs NPCs remains poorly investigated. Previous studies reported inconsistent results regarding the roles of JNK1 and JNK2, likely reflecting differences in experimental models of liver diseases and the complex, context-dependent interactions between the different hepatic cell populations during disease progression.^13^^,^^28^

In the current study, we used both systemic and cell-specific approaches in deleting JNK isoforms. We show that JNK plays a central and multifunctional role in regulating BA homeostasis as well as inflammatory response and liver injury in cholestatic liver disease. In particular, we provide evidence that JNK2 in NPCs, rather than BM-derived cells, is a key driver of immune cell infiltration by controlling the inflammatory milieu, whereas JNK1 and JNK2 in hepatocytes cooperatively maintain BA balance under cholestatic stress.

As reported for cholestasis, an elevated BA pool acts as an endogenous ligand for FXR activation, a negative feedback regulator of CYP7A1, to restrict de-novo BA synthesis via a JNK-dependent mechanism.^27^^,^^29^, ^30^, ^31^ Following BDL, combined deletion of *Jnk1* and *Jnk2* in hepatocytes (*Jnk1*^*Δhepa*^*/2*^*-/-*^) promoted a prominent accumulation of total and conjugated BA. This indicated that JNK1 and JNK2 are synergistically involved in hepatocyte-mediated BA synthesis and transport, supporting previously reported findings.^26^

Interestingly, TβMCA, a well-known endogenous FXR antagonist that suppresses FXR activity,^32^ was found to be increased in JNK-deficient livers, thus disrupting the hepatic FXR-CYP7A1 axis that regulates BA synthesis. Correspondingly, we found a reduction in *Fxr* mRNA expression in JNK-deficient livers, whereas *Lrh1* and *Tgr5*—both FXR-target genes^33^—were elevated. These findings suggest that TβMCA accumulation antagonizes FXR and delays its feedback suppression of BA synthesis, as evidenced by the marked upregulation of CYP7A1 protein, particularly in *Jnk1*^*Δhepa*^*/2*^*-/-*^ livers.

Moreover, dysregulation of BA synthesis was associated with a significant defect in the BA transport system into and out of hepatocytes in *Jnk1*^*Δhepa*^*/2*^*-/-*^ livers. Unexpectedly, we observed that *Bsep* was upregulated in *Jnk1*^*Δhepa*^*/2*^*-/-*^ and *Jnk2*^*-/-*^ livers compared with WT livers, whereas key efflux transporters such as *Mrp2*, *Mrp4*, and *Mdr2* were downregulated, indicating impaired biliary excretion. Notably, *Ntcp* and *Oatp*, major BA uptake transporters that are typically downregulated in cholestasis,^34^ were upregulated in *Jnk1*^*Δhepa*^*/2*^*-/-*^ livers, suggesting an enhanced reuptake of toxic BAs into hepatocytes, thus contributing to cellular stress. Collectively, these data demonstrate that JNK-deficient hepatocytes fail to maintain the balance of synthesis, uptake, and export of BA, thereby leading to exacerbated hepatic injury.

As observed in JNK-deficient mice, our analysis of liver tissue from human cholestatic patients, namely, primary sclerosing cholangitis (PSC) and primary biliary cholangitis (PBC), revealed a similar dysregulation of BA synthesis and transport, confirming previous studies.^9^, ^10^, ^11^^,^^35^ CYP7A1 expression was upregulated, whereas BA transporters BSEP, MRP2, and MDR3 were markedly downregulated compared with healthy controls at the protein level.

Interestingly, we recently reported an inverse correlation between JNK activation in hepatocytes and the extent of liver fibrosis in cholestatic patients, confirming their combined protective role in hepatocytes.^15^ These data suggest that the ablation of JNK signaling, particularly in hepatocytes, contributes to cholestasis progression by promoting BA accumulation and exacerbating hepatocellular damage and fibrosis.

BAs may induce cholestatic hepatic injury by triggering an inflammatory response.^36^^,^^37^ This hypothesis is supported by studies indicating that mitigating the inflammatory response led to reduced hepatic injury and fibrosis in cholestatic rodent models.^37^, ^38^, ^39^ Importantly, our findings showed enhanced inflammation and immune cell infiltration following BDL in mice lacking *Jnk2* in NPCs (*Jnk2*^*-/-*^ and *Jnk1*^*Δhepa*^*/2*^*-/-*^). Both genotypes displayed increased infiltrating CD45+, CD11b+, and F4/80+ immune cells. This was further supported by an elevated hepatic expression of proinflammatory cytokines *Il6, Il1β,* and Tgfβ, as well as chemokines and their receptors, including CCL5-CCR5 and CXCL1-CXCR2, both known to drive macrophage and neutrophil recruitment. Indeed, JNK has been implicated in immune cell infiltration, specifically in controlling macrophage polarization.^40^ Notably, Tnf-α and Tlr4, key mediators of immune activation in response to damage-associated molecular patterns (DAMPs), were significantly increased only in *Jnk1*^*Δhepa*^*/2*^*-/-*^ mice. This suggests an exclusive proinflammatory phenotype when JNK activity is interrupted in both hepatocytes and NPCs. Consequently, *Jnk2*^*-/-*^ mice exhibited enhanced ECM formation and portal fibrosis progression, which was further aggravated in *Jnk1*^*Δhepa*^*/2*^*-/-*^ livers. Collectively, these findings indicate that *Jnk2* in NPCs plays a crucial role in driving the inflammatory response and promoting hepatic fibrogenesis after BDL.

Previous studies revealed that *Jnk1* activation (eg, in Kupffer cells) is relevant during hepatic inflammation and could therefore contribute to liver fibrosis.^41^^,^^42^ In our study, it was unclear if increased inflammation, as found in *Jnk1*^*Δhepa*^/*2*^*-/-*^ mice after BDL, was mediated by loss of JNK2 in NPCs or BM-derived hematopoietic immune cells. To define the cellular origin of JNK2’s effects in NPCs, we performed BMT experiments. Our data strongly suggest that JNK2 in BM-derived cells (BMDCs) has no impact and is not the primary mediator of inflammation and fibrogenesis in this context. On the other hand, JNK2 in resident NPCs, including Kupffer cells and HSCs, most likely plays a crucial role in regulating immune cell recruitment and liver fibrogenesis in *Jnk1*^*Δhepa*^*/2*^*-/-*^ cholestatic mice.

Next, we sought to understand whether the increased cytokine response in cholestatic *Jnk2* knockout livers contributes to hepatic BA accumulation via dysregulation of BA control genes. Interestingly, we found a strong correlation between *Il6* mRNA expression and total BA content in *Jnk1*^*Δhepa*^/*2*^*-/-*^ mice livers. Notably, stimulation of human hepatocytes with IL6 or TNFα reduced the expression of BA transporters.^43^ Consistently, in vitro, IL6 suppressed BA-transporter expression in JNK-deficient hepatocytes, providing a molecular link between NPC-driven cytokine release and impaired BA homeostasis in JNK-deficient hepatocytes. At present, we cannot exclude that other cytokines (eg, TNFα and IL1β) might also contribute to our in vivo findings, although our data did not show a correlation between their mRNA expression and accumulation of BAs in *Jnk1*^*Δhepa*^/*2*^*-/-*^ livers.

In summary, cholestatic liver injury triggers a wound healing response associated with a sustained inflammatory response, in which hepatocytes and NPC cooperate to diminish the deleterious effects of hepatocellular damage. Absence of *Jnk1/2* in hepatocytes disrupts BA homeostasis and fuels BA overload, whereas *Jnk2* loss in NPCs aggravates the cholestatic inflammatory response and cytokine release that further suppress BA transporters and aggravate BA toxicity. Altogether, both mechanisms enhance cholestatic damage and accelerate fibrogenesis.

## Materials and Methods

### Generation of Mice

Mice were maintained and housed under pathogen-free conditions in a 12:12-hour light/dark cycle and with free access to food and water in compliance with the institutional guidelines. All mouse experiments have been approved by the German and Spanish legal authorities. *Alb-Cre* and global *Jnk2*-deficient (*Jnk2*^*−/−*^) mice were purchased from the Jackson Laboratory, whereas Roger J. Davis provided *Jnk1*^*LoxP/LoxP*^ and *Jnk2*^*LoxP/LoxP*^. *Jnk1*^*Δhepa*^*/2*^*−/−*^ (hepatocyte-specific deletion of JNK1 and global ablation of JNK2) mice were bred as previously reported.^17^^,^^44^

### Bile Duct Ligation

Obstructive cholestasis was induced by ligating the common bile duct for 4 weeks. Eight- to 10-week-old age-matched male mice on C57BL/6 genetic background (n = 5–7/group) were used according to the approved animal protocols (LANUV, Germany; No./AZ: 8402.04.2013.A184 and 84-02.04.2016.A080 and Spain; PROEX 125-1/20).

In brief, 8- to 10-week-old mice were narcotized using 0.01% Xylazin hydrochloride/1% Ketamin hydrochloride mixture, diluted in 0.9% NaCl. After reflex control and affirmation of narcotization, the abdominal wall was sterilized, then an abdominal midline skin incision was made through the abdominal musculature by a 1.5-cm long cut. The lobes of the liver are gently retracted to expose the bile duct. The bile duct is then carefully ligated with 2 pieces of sutures; one is dorsal, and the other is ventral to the bile duct. A small incision is used between both ligatures to ensure the blockage of the bile duct. The abdominal musculature and skin are closed with absorbable suture by a continuous fissure. Subsequently, mice were received Temgesic intraperiotoneally to avoid pain-induced stress. Control animals were opened and immediately closed (sham-operated).

### Transplantation of BMDCs and Construction of Jnk1^Δhepa^/2^−/−^ Chimeric Mice

We transferred BM from *Jnk1*^*Δhepa*^*/2*^*−/−*^ and WT mice into 7-week-old *Jnk1*^*Δhepa*^*/2*^*−/−*^ isogeneic recipients (*n* = 6–7 mice per group) after ablative γ-irradiation, as described previously.^16^^,^^45^ In brief, BMDCs were isolated from euthanized donor *Jnk1*^*Δhepa*^*/2*^*−/−*^ and WT mice. Recipient mice *Jnk1*^*Δhepa*^*/2*^*−/−*^ and WT were first irradiated using a cobalt irradiator and delivered a radiation dose of 6.8 G. Four hours later, the radiation process was repeated with a total dose of 13.6 G (split dose irradiation is used to limit the nonhematopoietic toxicity). Irradiated mice were injected within 18 hours after the second irradiation dose via the tail vein with donor BMDCs (1 × 10^6^) diluted in 100 μL of sterile phosphate buffered saline (PBS). Recipient mice are maintained in antibiotic-containing water for approximately 2 weeks. Finally, mice underwent BDL for 4 weeks.

### Human Samples

Human formalin-fixed and paraffin-embedded liver specimens were obtained from patients with PSC and PBC (n = 37 and n = 29, respectively) who underwent liver biopsy, resection, or transplantation. Control liver tissues without histologic signs of inflammation or fibrosis (n = 5) were obtained from individuals undergoing liver resection as a result of metastasis. The samples were collected retrospectively at the Institute of Pathology, RWTH Aachen University, Aachen, Germany; the Department of Pathology, Otto-von-Guericke University Magdeburg, Germany; the Institute of Pathology, Neuropathology and Molecular Pathology of the University of Innsbruck, Austria; and the tissue bank of the Pathology Institute of the University of Heidelberg under the approval of the local ethics committees (No. 61/18 Magdeburg; No 1115/2019 Innsbruck; No. EK166-12 Aachen; and No. S-043/2011 Heidelberg) as we previously reported.^15^

### Microarray Analysis

Affymetrix Microarray data have been deposited with the NCBI Gene Expression Omnibus (http://www.ncbi.nlm.nih.gov/geo/) under accession number GSE140498 as described previously.^46^

### Quantitative Real-time PCR

Total RNA from liver tissues was isolated using Trizol reagent (Invitrogen, Karlsruhe, Germany). cDNA was synthesized using Applied Biosystems High-Capacity cDNA Reverse Transcription Kit (4374966; Thermo Fisher Scientific). Relative gene expression was measured via real-time PCR using a Quant Studio 5 real-time PCR system (Thermo Fisher Scientific) and a SYBR Green PCR Kit (Invitrogen). *Gapdh* expression was used as an internal standard. Primer sequences are listed in Table 1.

**Table 1:** List of Primer Sequences for qRT-PCR

Gene	Forward primer	Reverse primer
*Apelin*	TCTTGGCTCTTCCCTCTTTTCA	GTGCTGGAATCCACTGGAGAA
*Acta2*	TGACAGAGGCACCACTGAACC	TCCAGAGTCCAGCACAATACCAGT
*ApelinR*	TCGGCTAAGGCTGCGAGTC	CGTCTGTGGAACGGAACAC
*Asbt*	GTCTGTCCCCCAAATGCAACT	CACCCCATAGAAAACATCACCA
*Bsep*	CTGCCAAGGATGCTAATGCA	CGATGGCTACCCTTTGCTTCT
*Ccl5*	TGCTGCTTTGCCTACCTCTC	TCCTTCGAGTGACAAACACGA
*Ccr5*	CACAGCATGGACAATAGCCAAGTACC	GCCATCTCTGACCTGCTCTTCC
*Co31a1*	TCCTGGTGGTCCTGGTACTG	AGGAGAACCACTGTTGCCTG
*Col1a1*	TGTGTGCGATGACGTGCAAT	GGGTCCCTCGACTCCTAC
*Cxcl1*	CCCAAACCGAAGTCATAGCCA	GTGCCATCAGAGCAGTCTGT
*Cxcr2*	TGGCATGCCCTCTATTCTGCC	CAAGGCTCAGCAGAGTCACC
*Cyp27a1*	GCCTCACCTATGGGATCTTCA	TCAAAGCCTGACGCAGATG
*Cyp7a1*	AGCAACTAAACAACCTGCCAGTACTA	GTCCGGATATTCAAGGATGCA
*Cyp8b1*	GGCTGGCTTCCTGAGCTTATT	ACTTCCTGAACAGCTCATCGG
*Fxr*	TCCAGGGTTTCAGACACTGG	GCCGAACGAAGAAACATGG
*Gapdh*	TCAAGCTCATTTCCTGGTATGAC	CTTGCTCAGTGTCCTTGCTG
*Hamp1*	CTCTGTTTTCCCACAACAGAC	TAGGGGAAGTGGGTGTCTC
*Il1β*	GCAGTGGTTCGAGGCCTAAT	CTCATCACTGTCAAAAGGTGGC
*Lrh1*	TTGAGTGGGCCAGGAGTAGT	ACGCGACTTCTGTGTGTGAG
*Il6*	ACTTCACAAGTCGGAGGCTT	TGCAAGTGCATCATCGTTGT
*Mdr1*	CTGTTGGCGTATTTGGGATGT	CAGCATCAAGAGGGGAAGTAATG
*Mdr2*	GCAGCGAGAAACGGAACAG	GGTTGCTGATGCTGCCTAGTT
*Mrp2*	TTCTGGGGAAAGGCACCATC	CCTTCAGGTCCCGAATGCTT
*Mrp3*	CACCATCAGCTCGGCTACAT	CAGGTCCACCCATGAGACAC
*Mrp4*	AGGAGCTTCAACGGTACTGG	GCCTTTGTTAAGGAGGGCTTC
*Msln*	CCCATCGAAGTGGTCAGTCTC	GGTGTATGACGGTCAGCTTAGA
*Muc1*	CTGTTCACCACCACCATGAC	CTTGGAAGGGCAAGAAAACC
*Ntcp*	CAAACCTCAGAAGGACCAAACA	GTAGGAGGATTATTCCCGTTGTG
*Oatp1*	GATCCTTCACTTACCTGTTCAA	CCTAAAAACATTCCACTTGCCATA
*Saa1*	CACCGGCTGCTCAAAGGGGTCCCGG	AAACCCGGGACCCCTTTGAGCAGCC
*Saa2*	TGATGCTGCCCAAAGG	GCCAGGAGGTCTGTAGTAA
*Saa3*	GGGTCTAGAGACATGTGGCG	TCTGGCATCGCTGATGACTT
*Tff3*	TTGCTGGGTCCTCTGGGATAG	TACACTGCTCCGATGTGACAG
*Tgfβ1*	CAACCCAGGTCCTTCCTAAA	GGAGAGCCCTGGATACCAAC
*Thy1*	GCTCTCAGTCTTGCAGGTGTC	CAGGCGAAGGTTTTGGTTCA
*Tlr4*	GCTTTCACCTCTGCCTTCAC	GAAACTGCCATGTTTGAGCA
Tnfα	ACTGAACTTCGGGGTGATCG	GCCATTTGGGAACTTCTCATCC
*Trg5*	CCTGGAACTCTGTTATCGCTCA	GCACTCGTAGACACCTTTGGG

qRT-PCR, quantitative real-time polymerase chain reaction.

### Histologic Evaluation and IF Staining

Hepatic tissue was fixed in 4% paraformaldehyde (PFA) immediately after extraction, embedded in paraffin, sectioned and stained for H&E or SR. Additionally, the hydroxyproline contents were measured colorimetrically as described previously.^47^ Samples were analyzed the degree of liver injury. The percentage of SR area fraction in all animals was quantified on 10 or 20 low-power (magnification, ×10) fields per slide, using NIH. The captured images were analyzed using Fiji (ImageJ) software (ImageJ Wiki). For IHC staining of PFA samples, liver sections were deparaffinized and rehydrated on slides. Antigen retrieval was performed in 10 mM Citrate buffer (pH 6) for 10 minutes. After washing the slides in PBST, they were exposed to 3% H_2_O_2_ for 10 minutes at 4°C and washed again. Nonspecific sites were blocked with blocking solution (PBS, 0.1% bovine serum albumin [BSA], 0.2% TritonX-100) for 30 minutes at room temperature (RT). Primary antibodies were diluted in PBT (PBS, 0.1% BSA, 0.2% TritonX-100) at 4°C in a humid box, and the staining was performed at 4°C overnight. The primary antibodies were used in the following dilutions: CD45 (70257; 1:400 dilution; Cell Signaling Technology), Cleaved caspase-3 (9661; 1:400 dilution; Cell Signaling Technology), Ki-67 (12202; 1:800; Cell Signaling Technology), and Muc-1 (ab15481; 1:200; Abcam). Slides were again washed in PBST and incubated with secondary antibodies in biotinylated secondary antibody solutions (1:200 diluted in PBT) for 1 hour at RT. The signal was developed with diaminobenzidine (DAB; SK4105, peroxidase substrate kit, Vector). Afterwards, the sections were counterstained using hematoxylin (MHS32; Merck Millipore) and then washed, dehydrated, and mounted with coverslips using the Roti Histokit (6638.1; Carl Roth). All bright-field images were analyzed and documented using an Axio Imager Z1 together with Axiovision software (Carl Zeiss).

For the IF staining, frozen cryosections were fixed in 4% formaldehyde, followed by washing in PBS; then the nonspecific binding was blocked with 2% BSA in 0.02 sodium azide for 1 hour at RT. Next, samples were incubated with the primary antibodies using the following dilutions: CK-19 (Ab52625; 1:200 dilution; Abcam), CD11b (550282; 1:200 dilution; BD), F4/80 (MCA497; 1:200 dilution; Bio-Rad laboratories), αSMA (Ab5694; 1:200 dilution; Abcam), and Col I (2150-1410; 1:200 dilution; Bio-Rad Laboratories) for 2 hours at RT. After washing, the slides were incubated with fluorescence-labeled secondary antibodies (Alexa Fluor 488; A21208 or Alexa Fluor 546; A11010; Invitrogen) at 1:500 dilution for 1 hour at RT. Finally, cell nuclei were counterstained using VECTASHIELD Antifade Mounting Medium with DAPI (H-1200; Vector Laboratories). All fluorescence-labeled cryosections were analyzed and documented using an Axio Imager Z1 fluorescence microscope together with Axiovision software (Carl Zeiss).

### Immunoblot Analysis

Liver tissues were homogenized in ice-cold NP40-Buffer containing 50 mM Tri-HCl (pH 7.5), 150 mM NaCl, 0.5% NP-40, and 50 mM NaF freshly supplemented with Complete Mini (Roche), PhosSTOP (Roche), 1 mM orthovanadate, and 1 mM Pefablock. Protein concentrations were determined by BIO-RAD protein assay (Bio-Rad Laboratories). Samples were separated by sodium dodecyl sulfate–polyacrylamide gel electrophoresis (SDS-PAGE), transferred to a cellulose membrane, and probed overnight with antibodies for CYP7A1 (PA5-100892; 1:1000 dilution; Invitrogen), BSEP (PA5-78690; 0.5 μg/mL dilution; Invitrogen), MRP2 (PA5-86719; 1:1000 dilution; Invitrogen), ABCB4/MDR2 (PA5-78692; 1:1000 dilution; Invitrogen), and GAPDH (MCA4739; 1:5000; Bio-Rad Laboratories). On the next day, the membrane was washed and probed with secondary antibodies HRP-linked anti-rabbit IgG (7074; 1:5000; Cell Signaling), and HRP-linked anti-mouse IgG (sc-516102; 1:5000; Santa Cruz Biotechnology).

### Isolation and Culture of Hepatocytes

Primary hepatocytes were isolated from the livers of 8- to 12-week-old mice as previously described.^48^ In brief, a cell suspension of hepatocytes and NPCs was obtained from mouse liver by the collagenase perfusion method. The digested liver was filtered and centrifuged at 50 × g for 2 minutes at 4°C. The pellet containing hepatocytes was washed and checked for viability. Hepatocytes from *Jnk*^*f/f*^*, Jnk2*^*Δhepa*^ and *Jnk1*^*Δhepa*^*/2*^*Δhepa*^ livers were cultured in collagen-coated 6-well plates at 5 × 10^5^ cells per well with Dulbecco’s modified Eagle’s medium (DMEM) supplemented with 10% fetal calf serum (FCS) and 1% penicillin/streptomycin in a humidified atmosphere containing 5% CO_2_ at 37°C. Four hours after seeding, the medium was replaced. On the next day, unattached cells were washed out, then the cells were treated with or without 50 ng/mL H-IL6 (8954-SR, R&D System) for 24 hours. Hepatocytes were harvested for RNA extraction.

### Statistical Analysis

All data are expressed as mean ± standard error of the mean (SEM). Statistical significance was determined by 1-way analysis of variance (ANOVA) followed by a Bonferroni multiple-comparison test. Comparisons of 2 groups were analyzed using an unpaired, 2-tailed Student *t*-test. *P* values less than .05 were considered significant.
